# Comprehensive systematic review and meta-analysis on the therapeutic efficacy of curcumin in osteoporosis: unveiling mechanisms and preclinical evidence

**DOI:** 10.3389/fnut.2025.1590256

**Published:** 2025-05-21

**Authors:** Sheng-lei Yang, Jing-xiang Wang, Fei-er Ma, Jiang-hua He, Ao Zhang, Xiao-ming Sun, Ying Wei, Yan Wang

**Affiliations:** ^1^School of Sports Medicine and Rehabilitation, Beijing Sport University, Beijing, China; ^2^Rehabilitation Medicine Center, Qilu Hospital of Shandong University Dezhou Hospital, Dezhou, China; ^3^Department of Rehabilition, West Coast New District Hospital of Chinese Medicine, Huangdao, Shandong, China

**Keywords:** osteoporosis, curcumin, animal model, preclinical study, meta-analysis, systematic review, mechanism

## Abstract

**Background:**

Osteoporosis (OP) is a common degenerative bone disease that seriously affects the quality of life of patients and poses a significant public health burden. Curcumin (CUR), a natural compound, has attracted much attention due to its anti-inflammatory, antioxidant and bone protective effects. However, there is currently a lack of systematic evaluation of the efficacy and mechanism of CUR in treating OP.

**Methods:**

This study is a systematic review and meta-analysis conducted per PRISMA guidelines. Studies meeting the inclusion criteria were retrieved and screened from the PubMed, Embase, Web of Science, and Cochrane Library databases. The included studies were limited to animal models of OP, and the intervention group was treated with a single dose of CUR. A meta-analysis was performed using Review Manager 5.4 and R Studio software. The standardized mean difference (SMD) and 95% confidence interval (CI) were calculated using the fixed-effect or random-effects model. Sources of heterogeneity, sensitivity, and publication bias were also explored.

**Results:**

A total of 17 high-quality studies involving 282 animals were included. The results of the metaanalysis showed that compared with the control group, CUR significantly increased bone mineral density (BMD of the femur: SMD = 2.18, 95% CI: 1.53–2.83; BMD of the tibia: SMD = 1.08, 95% CI: 0.30–1.87), improved the trabecular microstructure (BV/TV: SMD = 2.74, 95% CI: 1.84–3.64; Tb.N: SMD = 2.31, 95% CI: 1.65–2.96; Tb.Th: SMD = 2.09, 95% CI: 1.43–2.76; Tb.Sp: SMD = −2.32, 95% CI: −3.15 to −1.50). In addition, CUR significantly reduced serum CTX-1 and TRAP-5b levels, while increasing OCN and ALP levels. Mechanism studies have shown that CUR may act through OPG/RANKL, Wnt/β-catenin, NF-κB, MAPK, and TGF-β/Smad2/3 signaling pathways.

**Conclusion:**

This study is the first to systematically evaluate CUR's therapeutic effect on an OP animal model. The results show that CUR can significantly improve the pathological state of osteoporosis through a multi-target mechanism and has good therapeutic potential. However, heterogeneity and differences in the quality of the literature suggest that high-quality prospective studies are needed to verify the clinical value of CUR further.

## 1 Introduction

**Figure 1:**
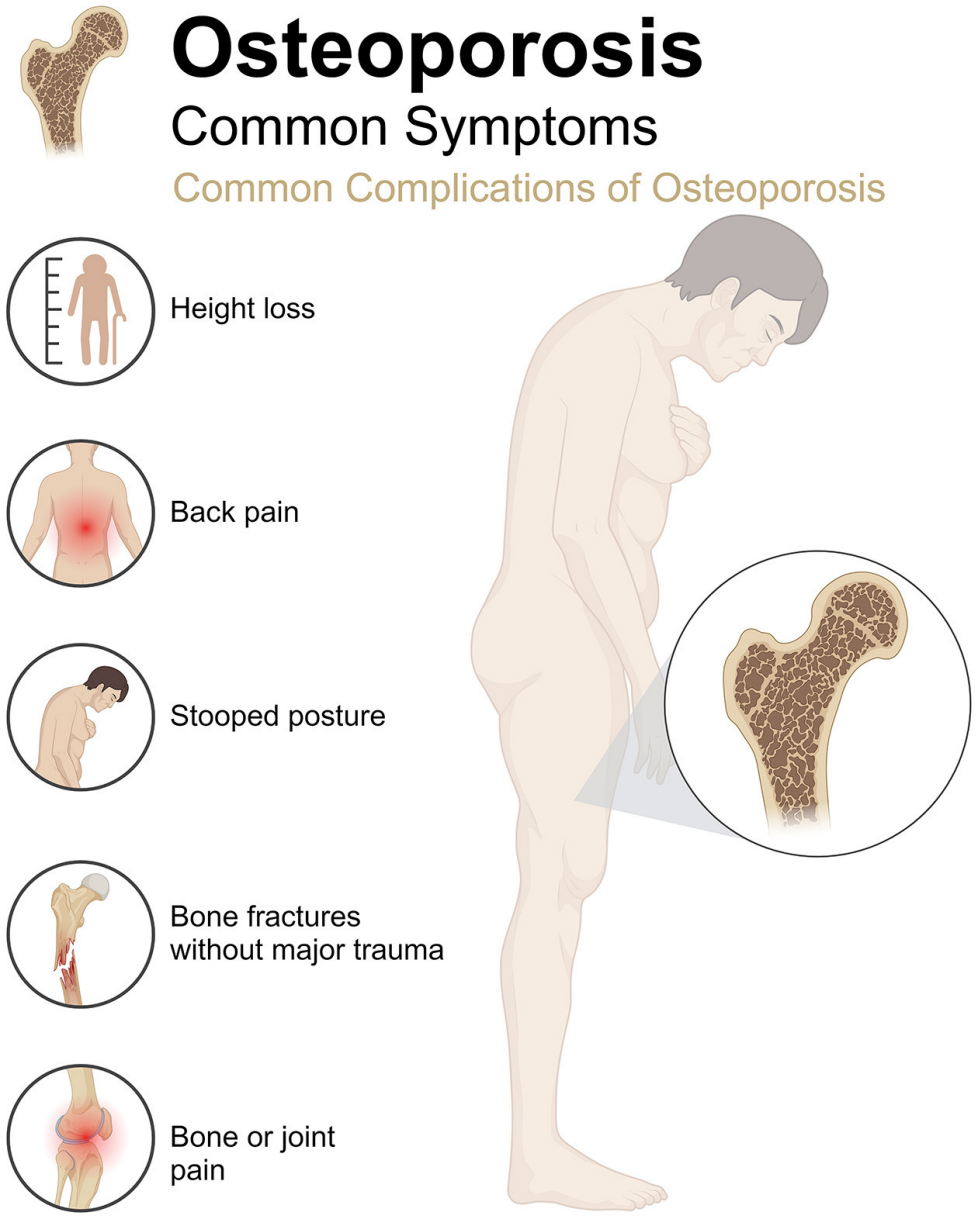
Clinical manifestations and common complications of osteoporosis.

Osteoporosis (OP) is a metabolic bone disease characterized by decreased bone mass, degradation of bone microstructure, and increased bone fragility (1). The core pathological mechanism is a dynamic imbalance in bone remodeling, where osteoclast bone resorption activity exceeds osteoblast bone formation capacity, decreasing in bone tissue quality and mechanical properties (2). This process significantly damages the bone structure and increases the risk of fracture (Figure 1). With the acceleration of global aging, the incidence of OP continues to rise (3). According to statistics, the prevalence rate of OP in women over 50 years old is 58%, while that in men is about 23%−35.6% (4, 5). In China, OP has become the third most common chronic disease after hypertension and diabetes, with a high incidence in postmenopausal women and the elderly (6). OP not only seriously affects the quality of life of patients but also imposes a heavy socioeconomic burden and is an important public health issue that requires urgent attention. From a pathological mechanism perspective, the occurrence of OP is closely related to the decreased differentiation ability of bone marrow mesenchymal stem cells (BMSCs) into osteoblasts and the disordered cell communication in the bone metabolism regulatory network (7). In addition, factors such as estrogen deficiency, insufficient calcium and vitamin D, and insufficient exercise significantly increase the risk of OP (8, 9). Currently, the conventional treatment strategies for OP mainly include anti-bone resorption drugs (such as bisphosphonates and selective estrogen receptor modulators) and drugs that promote bone formation (such as recombinant human parathyroid hormone) (10, 11). Although these treatment regimens can slow bone loss and reduce fracture risk to some extent, their long-term use is often limited by side effects (such as gastrointestinal discomfort and mandibular necrosis) and limited efficacy (12, 13). Therefore, developing new treatment strategies that are safe, effective, and have fewer side effects has become an important research direction.

**Figure 2:**
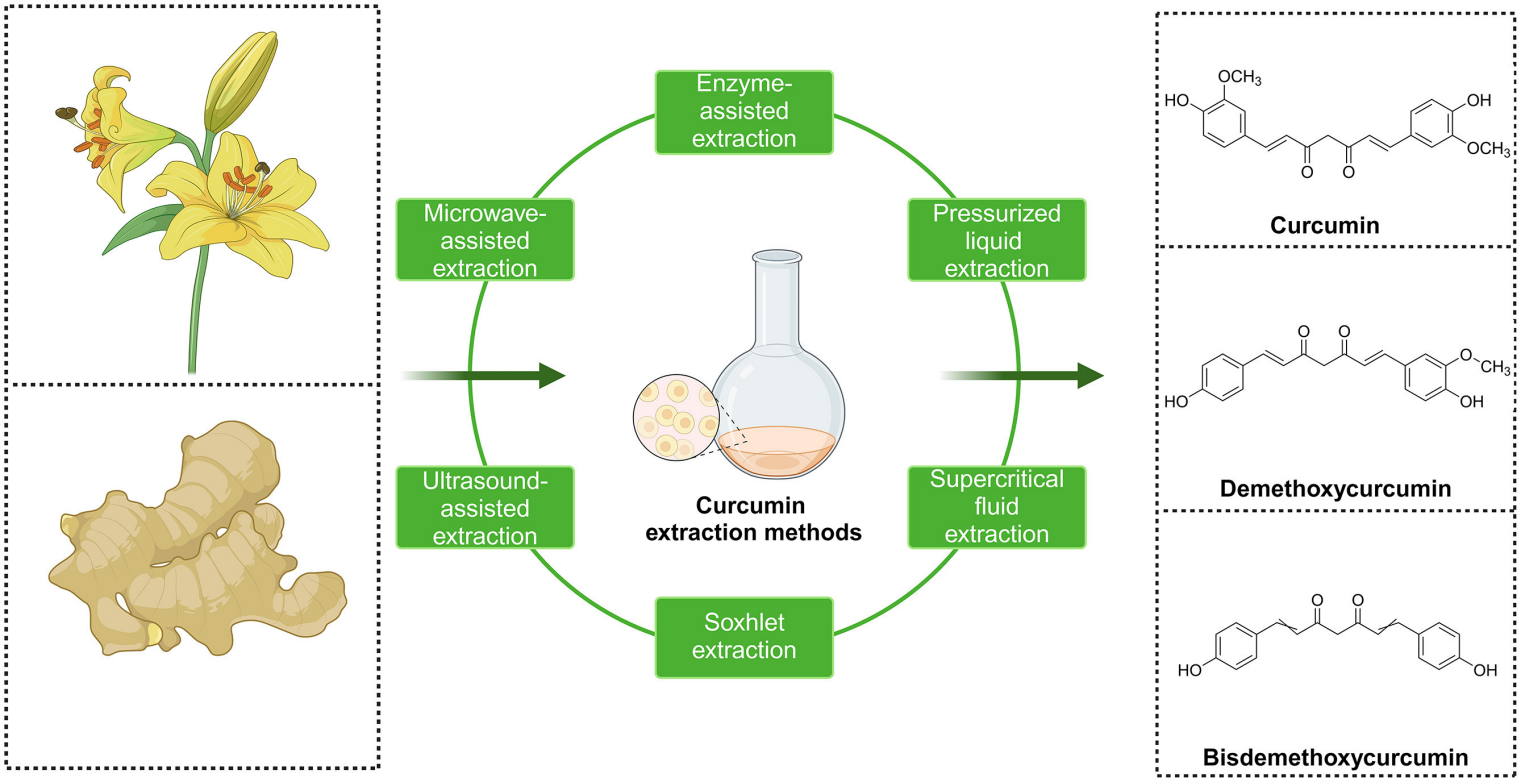
Three major chemical structures of curcumin compounds and main methods for extracting curcumin from plants.

Natural compounds have attracted much attention in disease prevention and treatment due to their advantages of being widely available, highly safe, inexpensive, and having few side effects, especially in OP treatment (14–17). Curcumin (CUR), a natural phenolic compound extracted from the rhizome of turmeric, is considered a potential drug candidate for the treatment of OP due to its anti-inflammatory, antioxidant, and anti-apoptotic biological activities (Figure 2) (18–20). CUR has been widely studied for the treatment of various diseases (e.g., cancer, cardiovascular disease, and metabolic diseases), and its pharmacological mechanisms are diverse and abundant. It has particularly shown significant advantages in regulating bone metabolism (21–23). CUR's anti-inflammatory and antioxidant properties are particularly critical for protecting bone metabolism. CUR can scavenge reactive oxygen species (ROS) and inhibit the release of inflammatory factors such as TNF-α and IL-6, thereby reducing oxidative stress and chronic inflammation damage to bone tissue (24, 25). In addition, CUR can also improve bone tissue's self-repair ability by improving mitochondria's oxidative state, increasing mitochondrial membrane potential, and improving osteoblast apoptosis induced by oxidative stress (26). Although the potential medicinal value of CUR in the treatment of OP has been verified in several animal experiments and cell studies, there is currently limited systematic evaluation and clinical evidence of its efficacy. Systematic reviews and meta-analyses based on preclinical studies not only help clarify the specific mechanisms and effects of CUR in treating OP but also provide key information for its clinical translation. This study comprehensively evaluates the therapeutic effects of CUR in animal models of OP by integrating preclinical evidence, focusing on its potential role in improving bone density, regulating bone metabolism, and protecting bone structure. At the same time, the meta-analysis will lay a theoretical foundation for the clinical application of CUR in the treatment of OP in the future and provide an important reference for the further development of natural compound therapies.

## 2 Methods

This research followed the guidelines set by the Preferred Reporting Items for Systematic Reviews and Meta-Analyses (PRISMA) and was registered on the PROSPERO platform of the International Systematic Reviews Registry (registration number CRD42024605225) (27).

### 2.1 Database and literature search strategies

This research performed a literature search across four databases: Pubmed, Embase, Web of Science, and the Cochrane Library, to gather experimental animal studies on the use of CUR for treating OP. The search involved the keywords “osteoporosis” and “curcumin.” The search period was from the establishment of the database to October 2024, and there were no restrictions on the language. The search approach combined subject keywords and free words, with adjustments made to fit the characteristics of each database, ensuring all relevant studies were captured. Table 1 summarizes the detailed search strategies for each database.

**Table 1:** Database search strategies.

**Database**	**Search Strategy**
PubMed	(“Curcumin”[Mesh]) OR (((((((Curcumin Phytosome) OR (Phytosome, Curcumin)) OR (1,6-Heptadiene-3,5-dione, 1,7-bis(4-hydroxy-3-methoxyphenyl)-, (E,E)-)) OR (Diferuloylmethane)) OR (Turmeric Yellow)) OR (Yellow, Turmeric)) OR (Mervia))) AND ((“Osteoporosis”[Mesh]) OR ((((((((((((((((((((((Osteoporoses) OR (Osteoporosis, Age-Related)) OR (Osteoporosis, Age Related)) OR (Age-Related Osteoporosis)) OR (Age-Related Osteoporoses)) OR (Age Related Osteoporosis)) OR (Osteoporoses, Age-Related)) OR (Bone Loss, Age-Related)) OR (Age-Related Bone Loss)) OR (Age-Related Bone Losses)) OR (Bone Loss, Age Related)) OR (Bone Losses, Age-Related)) OR (Osteoporosis, Senile)) OR (Osteoporoses, Senile)) OR (Senile Osteoporoses)) OR (Senile Osteoporosis)) OR (Osteoporosis, Involutional)) OR (Osteoporosis, Post-Traumatic)) OR (Osteoporosis, Post Traumatic)) OR (Post-Traumatic Osteoporoses)) OR (Post-Traumatic Osteoporosis)) OR (Bone loss)))
Web of science	TS = (Osteoporosis OR Osteoporoses OR Osteoporosis, Age-Related OR Osteoporosis, Age Related OR Age-Related Osteoporosis OR Age-Related Osteoporoses OR Age Related Osteoporosis OR Osteoporoses, Age-Related OR Bone Loss, Age-Related OR Age-Related Bone Loss OR Age-Related Bone Losses OR Bone Loss, Age Related OR Bone Losses, Age-Related OR Osteoporosis, Senile OR Osteoporoses, Senile OR Senile Osteoporoses OR Senile Osteoporosis OR Osteoporosis, Involutional OR Osteoporosis, Post-Traumatic OR Osteoporosis, Post Traumatic OR Post-Traumatic Osteoporoses OR Post-Traumatic Osteoporosis OR Bone loss) AND TS=(Curcumin OR Curcumin Phytosome OR Phytosome, Curcumin OR 1,6-Heptadiene-3,5-dione, 1,7-bis(4-hydroxy-3-methoxyphenyl)-, (E,E)- OR Diferuloylmethane OR Turmeric Yellow OR Yellow, Turmeric OR Meriva)
Embase	('decalcification, pathologic'/exp OR 'decalcification, pathologic' OR 'endocrine osteoporosis'/exp OR 'endocrine osteoporosis' OR 'osteoporotic decalcification'/exp OR 'osteoporotic decalcification' OR 'pathologic decalcification'/exp OR 'pathologic decalcification' OR 'osteoporosis'/exp OR 'osteoporosis') AND ('1, 7 bis (4 hydroxy 3 methoxyphenyl) 1, 6 heptadiene 3, 5 dione'/exp OR '1, 7 bis (4 hydroxy 3 methoxyphenyl) 1, 6 heptadiene 3, 5 dione' OR 'bis (4 hydroxy 3 methoxycinnamoyl) methane'/exp OR 'bis (4 hydroxy 3 methoxycinnamoyl) methane' OR 'curcumine'/exp OR 'curcumine' OR 'diferuloylmethane'/exp OR 'diferuloylmethane' OR 'nanocurc'/exp OR 'nanocurc' OR 'turmeric yellow'/exp OR 'turmeric yellow' OR 'curcumin'/exp OR 'curcumin')
Cochrane Library	(“Curcumin”[Mesh]) OR (((((((Curcumin Phytosome) OR (Phytosome, Curcumin)) OR (1,6-Heptadiene-3,5-dione, 1,7-bis(4-hydroxy-3-methoxyphenyl)-, (E,E)-)) OR (Diferuloylmethane)) OR (Turmeric Yellow)) OR (Yellow, Turmeric)) OR (Mervia))) AND ((“Osteoporosis”[Mesh]) OR ((((((((((((((((((((((Osteoporoses) OR (Osteoporosis, Age-Related)) OR (Osteoporosis, Age Related)) OR (Age-Related Osteoporosis)) OR (Age-Related Osteoporoses)) OR (Age Related Osteoporosis)) OR (Osteoporoses, Age-Related)) OR (Bone Loss, Age-Related)) OR (Age-Related Bone Loss)) OR (Age-Related Bone Losses)) OR (Bone Loss, Age Related)) OR (Bone Losses, Age-Related)) OR (Osteoporosis, Senile)) OR (Osteoporoses, Senile)) OR (Senile Osteoporoses)) OR (Senile Osteoporosis)) OR (Osteoporosis, Involutional)) OR (Osteoporosis, Post-Traumatic)) OR (Osteoporosis, Post Traumatic)) OR (Post-Traumatic Osteoporoses)) OR (Post-Traumatic Osteoporosis)) OR (Bone loss)))

### 2.2 Inclusion and exclusion criteria for literature

#### 2.2.1 Inclusion criteria

The inclusion criteria were formulated concerning the study object-intervention-control-outcome indicator-study type (PICOS) principle.

Study object (P): The study object was an OP animal model established by various methods (such as ovariectomy, drug induction, etc.).

Intervention (I): The experimental group received only single-drug CUR treatment without restrictions on the dose, administration method, duration, or drug type.

Control (C): The control group received a placebo or no treatment.

Outcome indicators (O): The primary outcome indicator was bone mineral density (BMD), including femoral BMD and tibial BMD. Secondary outcome indicators in-cluded: (1) Morphological indicators of bone microstructure: bone volume/total vol-ume (BV/TV), number of trabeculae (Tb.N), trabecular thickness (Tb.Th), trabecular separation (Tb.Sp); (2) serum bone turnover markers: osteocalcin (OCN), type I colla-gen potent carboxylic peptide (CTX-1), tartrate-resistant acid phosphatase 5b (TRAP-5b), serum calcium, and phosphorus.

Study type (S): This type of study is a controlled trial with a protocol that includes a control group and an experimental group.

#### 2.2.2 Exclusion criteria

The exclusion criteria are as follows: (1) reviews, meta-analyses, conference abstracts, case reports, *in vitro* studies, and clinical trials. (2) Studies in which CUR was combined with other compounds. (3) Studies in which CUR analogs were used. (4) Studies in which no OP or bone loss model was established. (5) Studies with incomplete data or lacking valid outcome indicators. (6) Studies that were withdrawn.

### 2.3 Data extraction

Two researchers independently used Endnote X9 software to manage and screen the literature. After removing duplicate literature, the retrieved articles' titles, ab-stracts, and full texts were screened. When there were disagreements or inconsistencies in the screening results, a third researcher was consulted to resolve the issue. After the screening was completed, the following data were collected using a standardized form for the included studies: (1) basic information, including the name of the first author, year of publication, animal species, age, weight, sample size, method of modeling with OP, and treatment of the control group; (2) basic information on the intervention, including the therapeutic dose of CUR and the duration of the intervention; and (3) primary outcome indicators, secondary outcome indicators, and outcome measurement data. The mean, standard deviation (SD), and number of animals for each indicator were extracted for comparison. If there were inconsistencies in the units of the outcome indicators, all non-international units were converted to international units; if a study included multiple intervention groups, only the control group and the data using CUR as the intervention were included; if a study included two or more different dose groups, all dose groups were included; if the outcome indicators were measured at different time points, the data obtained from the last time point measurement were used.

### 2.4 Data extraction

The quality of the literature included in this metaanalysis was evaluated using the SYRCLE (Systematic Review Centre for Experimental Laboratory Animal) tool for assessing the risk of bias (28). The SYRCLE risk of bias assessment tool for animal experiments has 10 items, including ① random sequence generation; ② baseline characteristic balance; ③allocation concealment; ④random placement of animals; ⑤ blinded experimental design; ⑥ random selection of outcome assessment; ⑦ blinded outcome assessment; ⑧ incomplete data processing; ⑨ selective outcome reporting; and ⑩ other potential bias. Each item is judged as having a “low risk of bias,” “high risk of bias,” or “unclear risk of bias.”

### 2.5 Statistical analysis

This study extracted data from the literature, finally included it, and imported it into Review Manager 5.4 and R Studio software for meta-analysis. First, the Q test and *I*^2^ statistic were employed to evaluate the heterogeneity across the studies. If *I*^2^ ≤ 50% or *P* ≥ 0.1, the studies are considered less heterogeneous, and the fixed-effect model is used; if *I*^2^ > 50% or *P* < 0.1, the studies are considered heterogeneous, and the random-effects model is used for analysis. Subgroup and meta-regression analyses were performed to explore further the potential sources of heterogeneity (such as intervention dose, intervention time, modeling method, etc.) when the number of studies exceeded 10. Sensitivity analyses were performed by sequentially excluding individual studies. In addition, publication bias was assessed using the Egger test and funnel plot analysis, and the potential impact of publication bias on the results was explored using quantitative and qualitative methods. This study analyzed publication bias for outcome indicators such as femoral BMD, BV/TV, Tb.N, Tb.Th, and Tb.Sp. The Trim-and-Fill approach was applied to adjust for potential bias and improve the reliability of the findings. The outcome indicators in this study were all continuous data, and the results were statistically analyzed using the standardized mean difference (SMD) and its 95% confidence interval (CI). The combined effect of each indicator was analyzed by drawing a forest plot. Considering that the differences in animal populations and experimental designs (different CUR doses, intervention times, modeling methods, and detection methods) might influence the reliability of the conclusions, so subgroup analyses were conducted based on these factors.

## 3 Results

### 3.1 Results of literature screening

**Figure 3:**
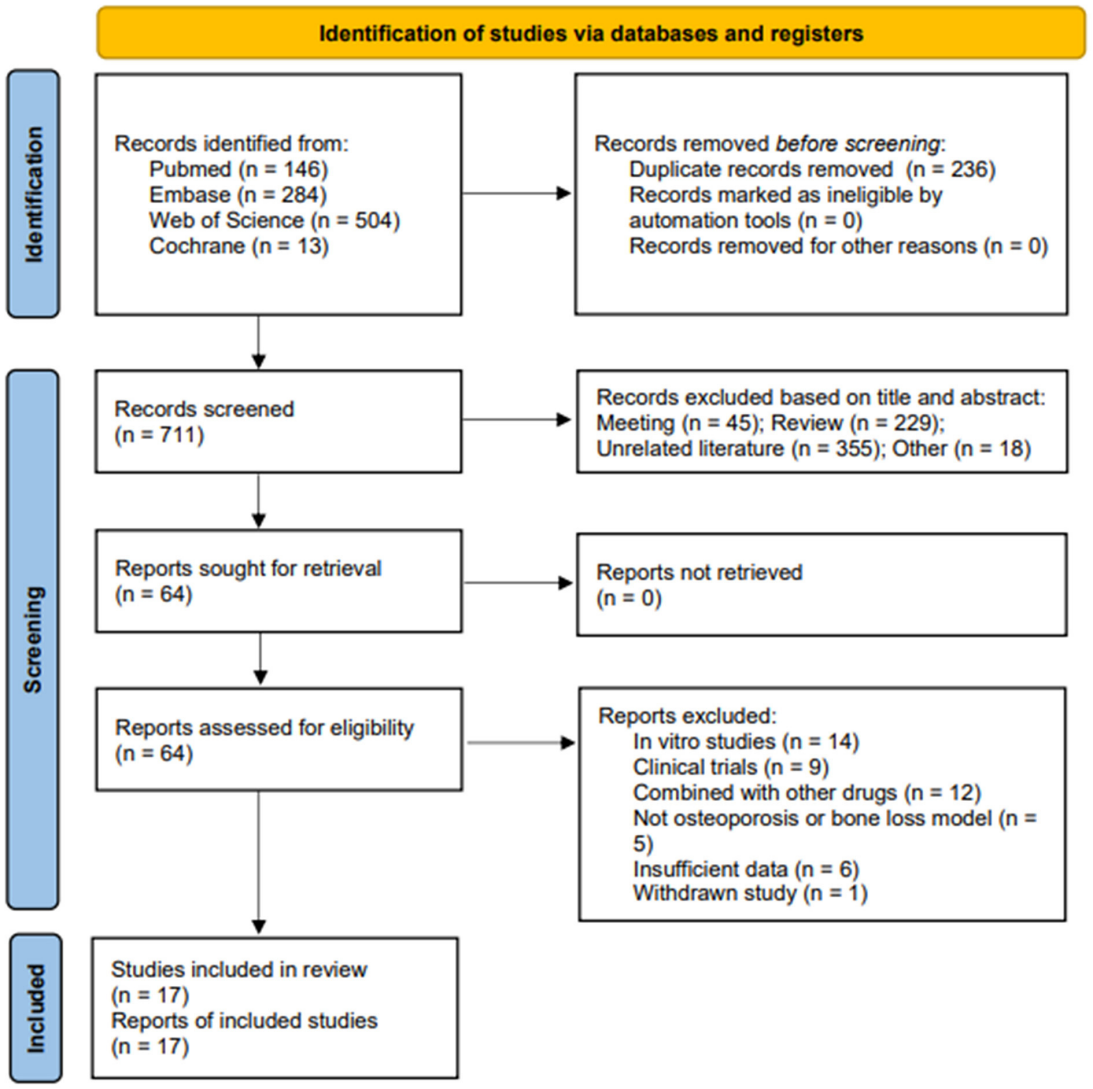
Flow chart of literature search and study selection. Initial searches retrieved 947 records from PubMed (*n* = 146), Web of Science (*n* = 504), Embase (*n* = 284), and Cochrane (*n* = 13). After removing 236 duplicates, 711 records underwent title/abstract screening. Studies were excluded if they were meeting abstracts (*n* = 45), reviews (*n* = 229), or unrelated literature (*n* = 355), leaving 64 full-text articles for eligibility assessment. A further 64 reports were excluded due to reasons such as being *in vitro* studies (*n* = 14), clinical trials (*n* = 9), combined with other drugs (*n* = 12), not being osteoporosis or bone loss models (*n* = 5), insufficient data (*n* = 6), and withdrawn studies (*n* = 1). Ultimately, 17 studies were included in the final review.

According to the search strategy, 947 potentially relevant documents were identified. After removing duplicate documents (*n* = 236), 711 documents were obtained. After screening based on reading the title or abstract, 647 irrelevant documents were excluded, and the remaining 64 were reviewed in full text. Forty-seven articles were excluded based on the exclusion criteria, including 14 *in vitro* cell experiments, nine clinical trials, five articles using models that did not meet the inclusion requirements, 12 articles in which the intervention was CUR combined with other drug treatments, six articles for which valid data could not be provided, and 1 article that the editor withdrew. Therefore, a total of 17 articles were included in the final meta-analysis (29–45). The process of literature search and selection is illustrated in Figure 3.

### 3.2 General characteristics of the included studies

This study included 17 animal studies on CUR treatment of osteoporosis. All studies were published between 2011 and 2024, and 282 animals participated in this study, with 151 in the treatment group and 131 in the control group. In terms of animal species, SD rats, Wistar rats, C57BL/6J mice, and BALB/c mice were used in the 17 studies. Among the models of OP, eight studies used the primary osteoporosis model established by ovariectomy (OVX) (29, 31, 32, 37–39, 41, 45), and nine studies used the secondary osteoporosis model established by drug injection (glucocorticoids, streptococci), gene knockout and hindlimb suspension, respectively, to establish a model of secondary osteoporosis (30, 33–36, 40, 42–44). The dose of CUR varied widely, with the minimum dose being 5 mg/kg/day and the maximum dose being 600 mg/kg/day. The duration of CUR administration ranged from 3 weeks to 15 weeks. Table 2 summarizes and lists the essential characteristics of the 17 included articles.

**Table 2:** Characteristics of the included studies in the meta-analysis.

**Study**	**Species**	**Age**	**Weight (g)**	**Model (method)**	**Sample size**		**Intervention**		**Duration**	**Outcome index**
					**Curcumin**	**Control**	**Curcumin**	**Control**		
Xin et al. (34)	SD rats	8 weeks	NR	DOP	12	12	40 mg/kg/day	Palm oil	6 weeks	BMD, BV/TV, Tb.N, Tb.Th, Tb.Sp
Chen et al. (35)	SD rats	NR	NR	GIOP	6	6	100 mg/kg/day	0.5% CMC-Na	60 days	BMD, CTX-1
Chen et al. (36)	SD rats	5 months	NR	GIOP	6	6	100 mg/kg/day	0.5% CMC-Na	60 days	BMD, CTX-1, OCN
Yang et al. (30)	B6C3-Tg 85Dbo/J mice	3 months	20 ± 1	GKIOP	9	9	600 mg/kd/day	NR	3 months	BMD, BV/TV, Tb.N, Tb.Th, Tb.Sp
Li et al. (33)	C57BL/6J mice	10 weeks	NR	GIOP	10	10	200 mg/kg/day	NR	12 weeks	BV/TV, Tb.N, Tb.Th, Tb.Sp, TRACP-5b, OCN, CTX-1, Ca
Xu et al. (45)	BALB/c mice	8 weeks	20.52 ± 1.27	OVX	10/10	10	5, 15 mg/kg/day	Physiological saline (containing DMSO)	8 weeks	BMD, BV/TV, Tb.N, Tb.Th, Tb.Sp
Fan et al. (42)	C57BL/6J mice	6 weeks	NR	DMOP	6	6	100 mg/kd/day	NR	8 weeks	BMD, BV/TV, Tb.N, Tb.Th, Tb.Sp
Hussan et al. (31)	SD rats	3 months	200–250g	OVX	8	8	110 mg/kd/day	Palm oil	60 days	BV/TV, Tb.N, Tb.Th, Tb.Sp
Kim et al. (29)	C57BL/6J mice	6 weeks	NR	OVX	7	7	9.5 mg/kg/day	PBS	8 weeks	BMD, BV/TV, Tb.N, Tb.Sp, CTX-1
Liang et al. (40)	SD rats	8 weeks	180 ± 20	DMOP	10	10	110 mg/kg/day	1% CMC-Na	8 weeks	BV/TV, Tb.N, Tb.Th, Tb.Sp, OCN, CTX-1
Jiang et al. (41)	SD rats	6 months	350–390	OVX	8	8	110 mg/kg/day	0.5% CMC-Na	12 weeks	BV/TV, Tb.N, Tb.Th, Tb.Sp
Ke et al. (44)	C57BL/6J mice	12 weeks	NR	GKIOP	6	6	200 mg/kg/day	NR	4 weeks	BMD, BV/TV, Tb.N, Tb.Th, Tb.Sp
Ke et al. (39)	SD rats	3 months	200–220	OVX	8	8	110 mg/kg/day	PBS	60 days	BV/TV, Tb.N, Tb.Sp, TRACP-5b,
Jiang et al. (38)	SD rats	6 months	350–390	OVX	10	10	110 mg/kg/day	0.5% CMC-Na	12 weeks	BMD, BV/TV, Tb.N, Tb.Th, Tb.Sp, Ca, P
Heo et al. (32)	C57BL/6J mice	10 weeks	20	OVX	5	5	18 mg/kg/day	Physiological saline	9 weeks	BMD, BV/TV
Partoazar and Goudarzi (43)	Wistar rats	NR	210 ± 20	DMOP	7	7	25 mg/kg/day	Physiological saline	3 weeks	Tb.N, Tb.Th, OCN, Ca, P,
Ahn et al. (37)	SD rats	6 months	280 ± 20	OVX	8	8	5 g/kg/day	PBS	15 weeks	BMD, BV/TV, Tb.N, Tb.Th, Tb.Sp, OCN, TRACP-5b, Ca, P

SD, Sprague-Dawley; NR, Not reported; DOP, Disuse osteoporosis; BMD, Bone Mineral Density; BV/TV, Bone Volume/Total Volume; Tb.N, Trabecular Number; Tb.Th, Trabecular Thickness; Tb.Sp, Trabecular Separation; GIOP, Glucocorticoid-Induced Osteoporosis; CMC-Na, Carboxymethyl Cellulose Sodium; CTX-1, C-terminal Telopeptide of Type I Collagen; OCN, Osteocalcin; GKIOP, Gene Knockout-Induced Osteoporosis; TRACP-5b, Tartrate-Resistant Acid Phosphatase Isoform 5b; Ca, Calcium; DMSO, Dimethyl Sulfoxide; OVX, Ovariectomy; DMOP, Diabetes Mellitus Osteoporosis; PBS, Phosphate Buffered Saline; P, Phosphorus.

### 3.3 Literature quality evaluation

**Figure 4:**
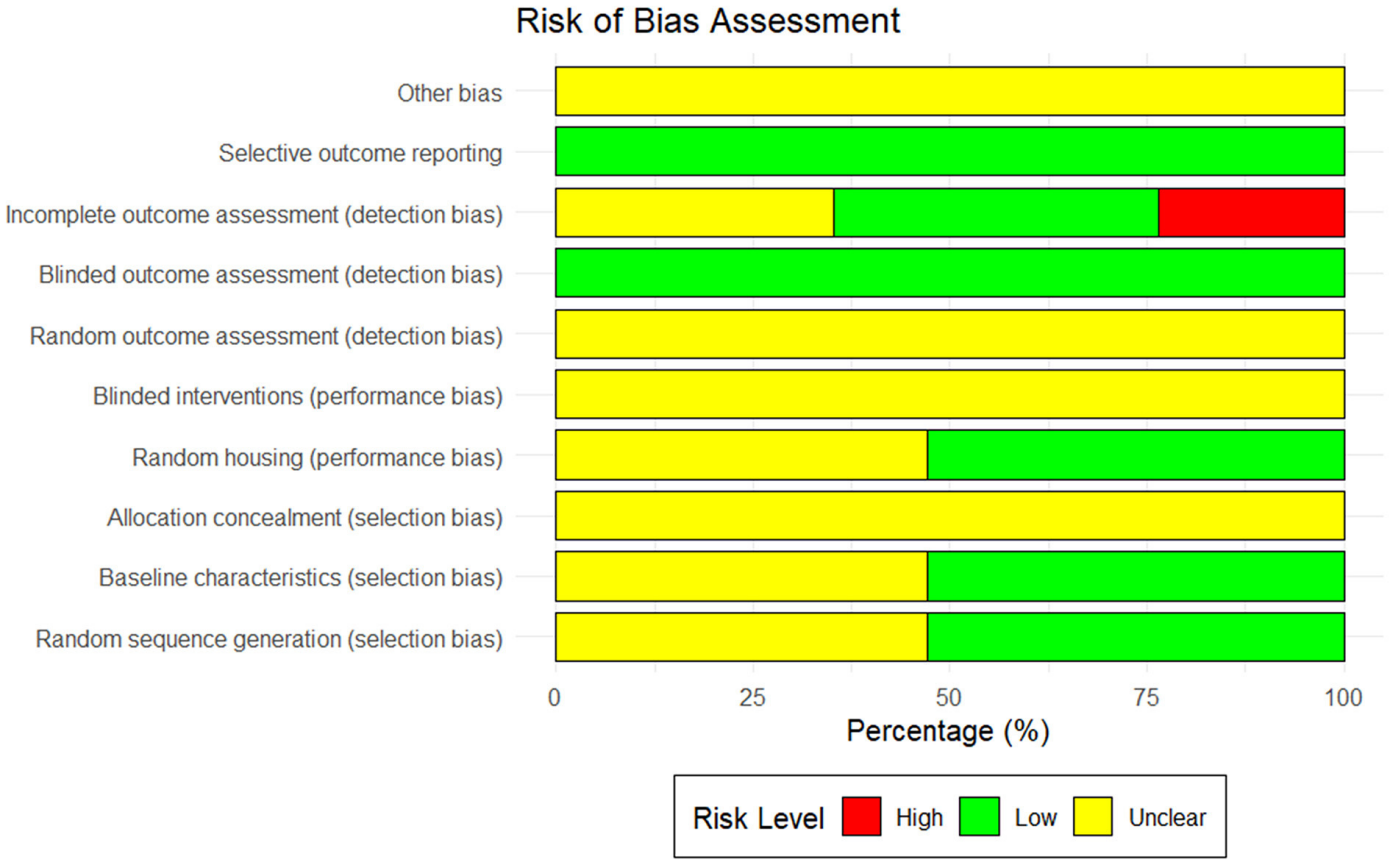
Risk of bias assessment for included studies.

Among the 17 studies included in this meta-analysis, the majority demonstrated an unclear risk of bias in areas like allocation concealment, random assignment, and random outcome assessment. Specifically, only two studies clearly used the random number table method for grouping (38, 45). Eight studies did not provide sufficient information to indicate whether the experimental animals were randomly assigned (29, 30, 35, 39, 40, 42–44); the other seven studies only indicated random grouping in the text without describing the specific grouping method (31–34, 36, 37, 41). In addition, regarding the assessment of incomplete data, six studies were shown to be at “unclear risk” (30, 31, 40, 42, 43, 45) for failing to provide sufficient details on the handling of missing data, and four other studies did not offer a detailed explanation of missing data (29, 33, 37, 39), which could result in potential reporting bias. The outcomes of the quality assessment of the included studies are shown in Figure 4.

### 3.4 Results of the meta-analysis

#### 3.4.1 BMD

##### 3.4.1.1 F-BMD

**Figure 5:**
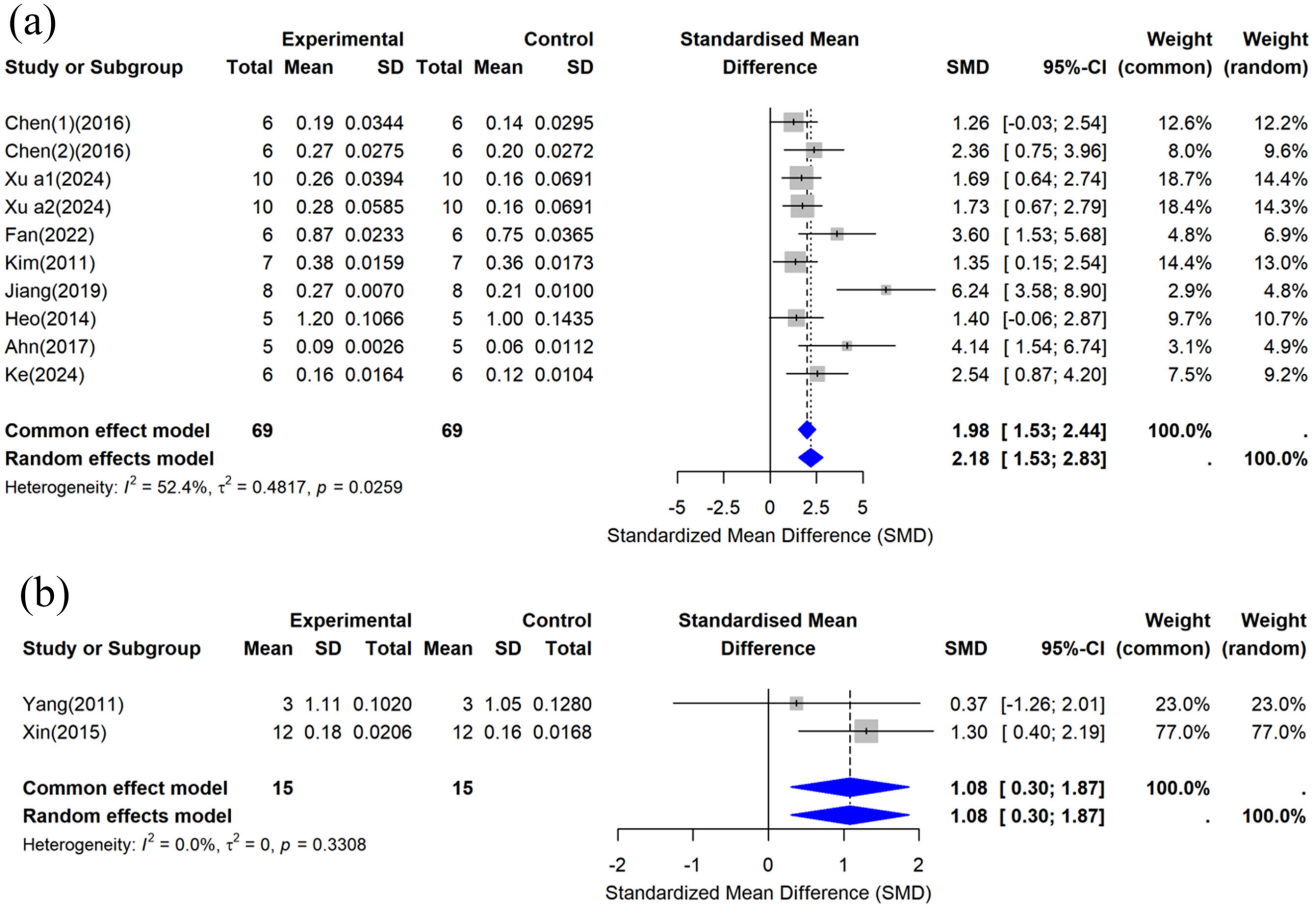
Forest plot of bone mineral density (BMD) after curcumin treatment. **(a)** Femoral BMD and **(b)** Tibial BMD.

This study included femoral BMD data from 10 studies (29, 32, 35–38, 42, 44, 45), and the combined analysis results are shown in Figure 5a. The results showed that compared with the control group, CUR could significantly increase femoral BMD (*n* = 138, SMD = 2.182, 95% CI: 1.529–2.83, *P* < 0.05). Heterogeneity analysis showed *I*^2^ = 52.4%, suggesting moderate heterogeneity (τ^2^ = 0.4817, *P* = 0.0259).

##### 3.4.1.2 T-BMD

This study included the tibial BMD data provided by two studies (30, 34), and the combined analysis results are shown in Figure 5b. The results showed that compared with the control group, CUR could significantly increase tibial BMD (*n* = 30, SMD = 1.0843, 95% CI: 0.2996–1.8690, *P* < 0.05). Heterogeneity analysis showed *I*^2^ = 0% (τ^2^ = 0, *P* = 0.3308), indicating no significant heterogeneity between studies.

#### 3.4.2 Bone microstructure parameters

##### 3.4.2.1 BV/TV

**Figure 6:**
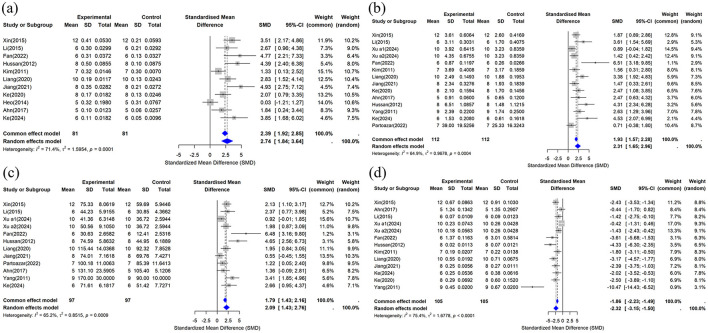
Forest plot of trabecular bone microstructure after curcumin treatment. **(a)** BV/TV, **(b)** Tb.N, **(c)** Tb.Th, and **(d)** Tb.Sp.

This study included BV/TV data from 11 studies (29, 31–34, 37, 39–42, 44), and the combined analysis results are shown in Figure 6a. The results showed that compared with the control group, the CUR intervention significantly improved BV/TV (*n* = 162, SMD = 2.7375, 95% CI: 1.8366–3.6384, *P* < 0.05). The heterogeneity analysis showed *I*^2^ = 71.4% (τ^2^ = 1.5954, *P* = 0.0001), indicating high heterogeneity among studies.

##### 3.4.2.2 Tb.N

This study included data on Tb.N from 14 studies (29–31, 33, 34, 37, 39–45), and the meta-analysis results are shown in Figure 6b. The results showed that compared with the control group, the CUR intervention significantly improved Tb.N (*n* = 224, SMD = 2.3094, 95% CI: 1.6548–2.9641, *P* < 0.05). The heterogeneity analysis showed *I*^2^ = 64.9% (τ^2^ = 0.9678, *P* = 0.0004), suggesting moderate heterogeneity among studies.

##### 3.4.2.3 Tb.Th

This study included Tb.Th data from 12 studies (30, 31, 33, 34, 37, 40–45). The results of the combined analysis are shown in Figure 6c. The results showed that the CUR intervention significantly increased Tb compared to the control group.Th (*n* = 194, SMD = 2.0915, 95% CI: 1.4264–2.7565, *P* < 0.05). Heterogeneity analysis showed *I*^2^ = 65.2% (τ^2^ = 0.8515, *P* = 0.0009), suggesting moderate heterogeneity among studies.

##### 3.4.2.4 Tb.Sp

Thirteen studies provided Tb.Sp data (29–31, 33, 34, 37, 39–42, 44, 45) that were included in this review. The combined results are shown in Figure 6d. The results showed that the CUR intervention significantly reduced Tb compared to the control group.Sp (*n* = 210, SMD = −2.3240, 95% CI: −3.1456 to −1.5024, *P* < 0.05). The heterogeneity analysis showed *I*^2^ = 75.4% (τ^2^ = 1.6778, *P* < 0.0001), indicating high heterogeneity among studies.

#### 3.4.3 Serum biochemical indicators

##### 3.4.3.1 Ca

**Figure 7:**
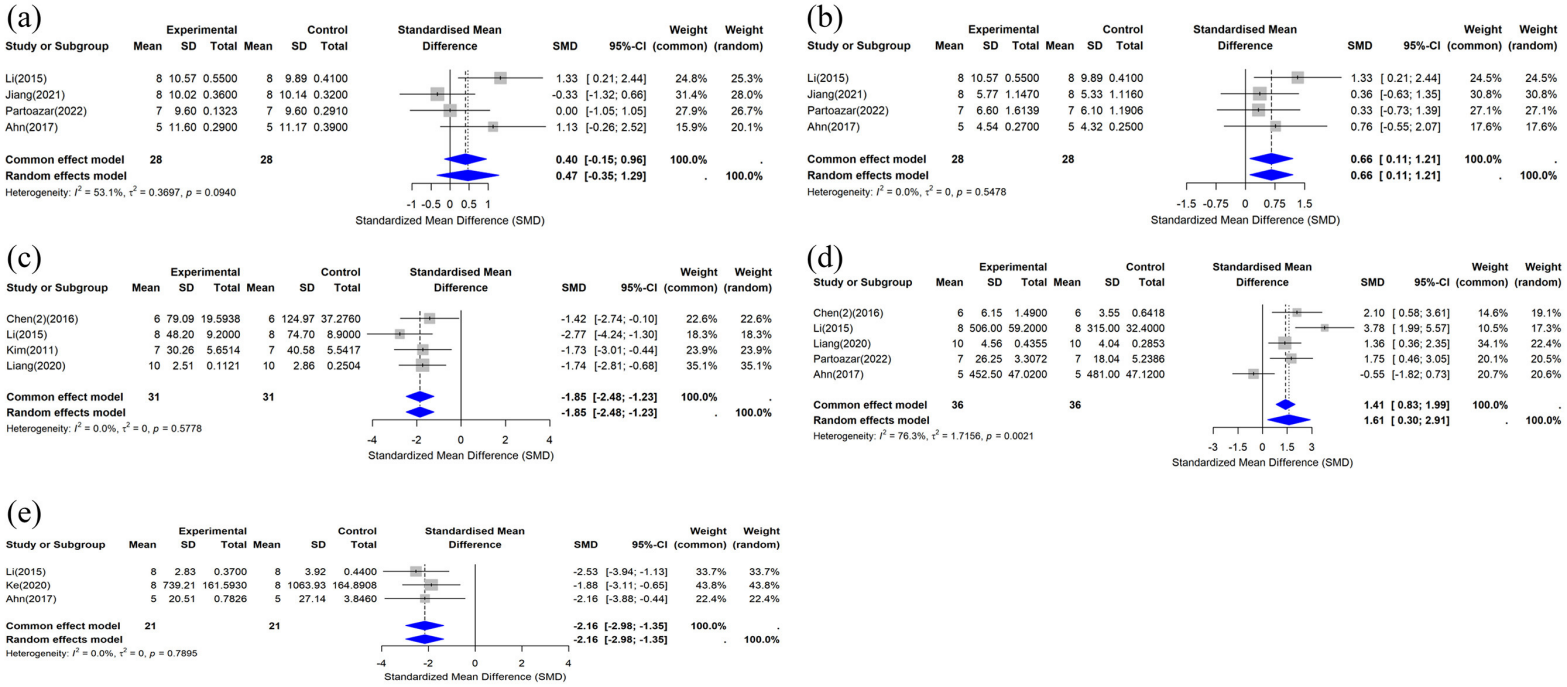
Forest plot of serum calcium, phosphorus, and biochemical markers after curcumin treatment. **(a)** Ca, **(b)** P, **(c)** CTX-1, **(d)** OCN, and **(e)** TRAP-5b.

This study included data on serum calcium concentration from four studies (33, 37, 41, 43), and the combined analysis results are shown in Figure 7a. Compared with the control group, the effect of the CUR intervention on serum calcium concentration was not statistically significant (*n* = 56, SMD = 0.4683, 95% CI: −0.3510 to 1.2877, *P* > 0.05). The heterogeneity analysis showed *I*^2^ = 53.1% (τ^2^ = 0.3697, *P* = 0.0940), indicating moderate heterogeneity.

##### 3.4.3.2 P

This study included data on serum phosphorus concentration from four studies (33, 37, 41, 43). The results of the combined analysis are shown in Figure 7b. The results showed that compared with the control group, the CUR intervention significantly increased serum phosphorus concentrations (*n* = 56, SMD = 0.6601, 95% CI: 0.1101 to 1.2100, *I*^2^ = 0%, *P* < 0.05). Heterogeneity analysis showed *I*^2^ = 0% (τ^2^ = 0, *P* = 0.5478), indicating no significant heterogeneity among the included studies.

##### 3.4.3.3 CTX-1

This study included data on serum CTX-1 concentrations from four studies (29, 33, 36, 40). The results of the combined analysis are shown in Figure 7c. The results showed that compared with the control group, the CUR intervention significantly reduced serum CTX-1 concentrations (*n* = 62, SMD = −1.8544, 95% CI: −2.4837 to −1.2252, *P* < 0.05). Heterogeneity analysis showed I^2^ = 0% (τ^2^ = 0, *P* = 0.5778), suggesting no significant heterogeneity between the included studies.

##### 3.4.3.4 OCN

This study included data on serum OCN concentrations from five studies (33, 36, 37, 40, 43), and the combined analysis results are shown in Figure 7d. The results showed that compared with the control group, the CUR intervention significantly increased serum OCN concentrations (*n* = 72, SMD = 1.6071, 95% CI: 0.3045 to 2.9098, *P* < 0.05). The heterogeneity analysis showed *I*^2^ = 76.3% (τ^2^ = 1.7156, *P* = 0.0021), indicating high heterogeneity between studies.

##### 3.4.3.5 TRAP-5b

This study included data on serum TRAP-5b concentrations from three studies (33, 37, 39). The results of the combined analysis are shown in Figure 7e. The results showed that compared with the control group, the CUR intervention significantly reduced serum TRAP-5b concentrations (*n* = 42, SMD, −2.1630, 95% CI: −2.9776 to −1.3484, *P* < 0.05). The heterogeneity analysis showed *I*^2^ = 0% (τ^2^ = 0, *P* = 0.7895), indicating no significant heterogeneity between studies.

### 3.5 Subgroup analysis

To explore the sources of heterogeneity in the included studies, subgroup analyses were performed for femoral BMD and trabecular-related indices (including BV/TV, Tb.N, Tb.Th, and Tb.Sp) to assess potential confounding factors that may have contributed to the high heterogeneity of the results (e.g., CUR dose, intervention duration, sample size, animal species, and detection method). The subgroup analysis results, shown in Table 3, indicated that treatment duration, CUR dose, sample size, animal species, and detection method were not confirmed as the primary sources of heterogeneity.

**Table 3:** Subgroup analysis of curcumin treatment on bone mineral density and trabecular microstructure.

**Groups**	**Categories**	**Number of studies included**	**Number of cases (EG/CG)**	**I^2^ (%)**	**Heterogeneity (P)**	**Pooling model**	**Z Test (P)**
**F-BMD**							
Animals species	SD rat	4	25/25	76.5	0.0051	Random	< 0.0001
	C57BL/6 mice	4	24/24	32.2	0.2193	Fixed	< 0.0001
	BALB/c mice	2	20/20	0	0.9537	Fixed	< 0.0001
Sample size	≤ 12	6	34/34	31.2	0.2017	Fixed	< 0.0001
	>12	4	35/35	73.5	0.0102	Random	< 0.0001
Intervention week	① ≤ 8 weeks	7	51/51	0	0.4911	Fixed	< 0.0001
	>8 weeks	3	18/18	81.7	0.0042	Random	< 0.0001
Curcumin dosage	① ≤ 60 mg/kg	5	37/37	0	0.4243	Fixed	< 0.0001
	②>60 mg/kg, ≤ 120 mg/kg	4	26/26	75.7	0.0063	Random	< 0.0001
	>180 mg/kg	1	6/6	-	-	Fixed	< 0.0001
Test method	CT	8	57/57	66.1	0.004	Random	< 0.0001
	X ray	2	12/12	24.0	0.251	Fixed	< 0.0001
Modeling method	POP	6	45/45	65.8	0.0122	Random	< 0.0001
	SOP	4	24/24	25.5	0.2584	Fixed	< 0.0001
**BV/TV**							
Animals species	SD rat	6	51/51	51.7	0.066	Random	< 0.001
	C57BL/6 mice	5	30/30	81.2	< 0.001	Random	< 0.001
Sample size	≤ 12	5	28/28	77.0	0.0016	Random	< 0.001
	>12	6	53/53	64.9	0.0142	Random	< 0.001
Intervention week	≤ 8 weeks	7	57/57	56.4	0.0323	Random	< 0.001
	> 8weeks	4	24/24	82.0	< 0.001	Random	< 0.001
Curcumin dosage	① ≤ 60 mg/kg	4	29/29	78.9	0.0026	Random	< 0.001
	② >60 mg/kg, ≤ 120 mg/kg	5	40/40	53.3	0.0731	Random	< 0.001
	③>120 mg/kg, ≤ 180 mg/kg	2	12/12	0	0.4025	Fixed	< 0.001
Modeling method	POP	6	41/41	78.2	< < 0.001	Random	< 0.001
	SOP	5	40/40	0	0.6211	Fixed	< 0.001
**Tb.N**							
Animals species	SD rat	6	51/51	44.2	0.1108	Fixed	< 0.001
	C57BL/6 mice	5	34/34	64.0	0.0254	Random	< 0.001
	BALB/c mice	2	20/20	0.0	0.4465	Fixed	< 0.001
	Wistar rat	1	7/7	-	-	Fixed	< 0.001
Sample size	≤ 12	3	17/17	53.8	0.1148	Random	< 0.001
	>12	11	95/95	62.2	0.0032	Random	< 0.001
Intervention week	≤ 8 weeks	10	84/84	72.1	0.0002	Random	< 0.001
	> 8 weeks	4	28/28	21.5	0.2814	Fixed	< 0.001
Curcumin dosage	① ≤ 60 mg/kg	5	39/39	0.0	0.4727	Fixed	< 0.001
	② >60 mg/kg, ≤ 120 mg/kg	6	52/52	66.3	0.0110	Random	< 0.001
	③ >120 mg/kg, ≤ 180 mg/kg	3	21/21	0	0.0889	Fixed	< 0.001
Modeling method	POP	7	56/56	50.2	0.0611	Random	< 0.001
	SOP	7	56/56	72.7	0.0012	Random	< 0.001
**Tb.Th**							
Animals species	SD rat	5	43/43	71.0	0.0080	Random	< 0.001
	C57BL/6 mice	3	18/18	59.5	0.0846	Random	< 0.001
	BALB/c mice	3	29/29	73.5	0.0229	Random	< 0.001
	Wistar rat	1	7/7	-	-	Fixed	< 0.001
Sample size	≤ 12	2	11/11	87.0	0.0056	Random	< 0.001
	>12	10	86/86	61.8	0.0050	Random	< 0.001
Intervention week	≤ 8 weeks	8	69/69	65.8	0.0046	Random	< 0.001
	> 8 weeks	4	28/28	71.1	0.0157	Random	< 0.001
Curcumin dosage	① ≤ 60 mg/kg	5	44/44	0.0	0.4227	Fixed	< 0.001
	② >60 mg/kg, ≤ 120 mg/kg	5	38/38	81.6	0.0002	Random	< 0.001
	③ >120 mg/kg, ≤ 180 mg/kg	2	15/15	0	0.3660	Fixed	< 0.001
Modeling method	POP	5	41/41	71.7	0.0070	Random	< 0.001
	SOP	7	56/56	49.5	0.0644	Fixed	< 0.001
**Tb.Sp**							
Animals species	SD rat	6	51/51	67.5	0.009	Random	< 0.0001
	C57BL/6 mice	4	25/25	20.0	0.290	Fixed	< 0.0001
	BALB/c mice	3	29/29	92.6	< 0.001	Random	< 0.0001
Sample size	≤ 12	4	23/23	58.1	0.0668	Random	< 0.0001
	>12	9	82/82	80.2	< 0.001	Random	< 0.0001
Intervention week	≤ 8 weeks	9	77/77	67.6	0.0018	Random	< 0.0001
	>8 weeks	4	28/28	87.5	< 0.001	Random	< 0.0001
Curcumin dosage	① ≤ 60 mg/kg	5	44/44	60.7	0.0376	Random	< 0.0001
	② >60 mg/kg, ≤ 120 mg/kg	5	40/40	0	0.4941	Fixed	< 0.0001
	③ >120 mg/kg, ≤ 180 mg/kg	3	21/21	89	< 0.001	Random	< 0.0001
Modeling method	POP	7	56/56	70.1	0.0027	Random	< 0.0001
	SOP	6	49/49	75.6	0.0010	Random	< 0.0001

F-BMD, Femoral Bone Mineral Density; BV/TV, Bone Volume/Total Volume; Tb.N, Trabecular Number; Tb.Th, Trabecular Thickness; Tb.Sp, Trabecular Separation; POP, Primary Osteoporosis; SOP, Secondary Osteoporosis.

### 3.6 Meta-regression analysis

**Figure 8:**
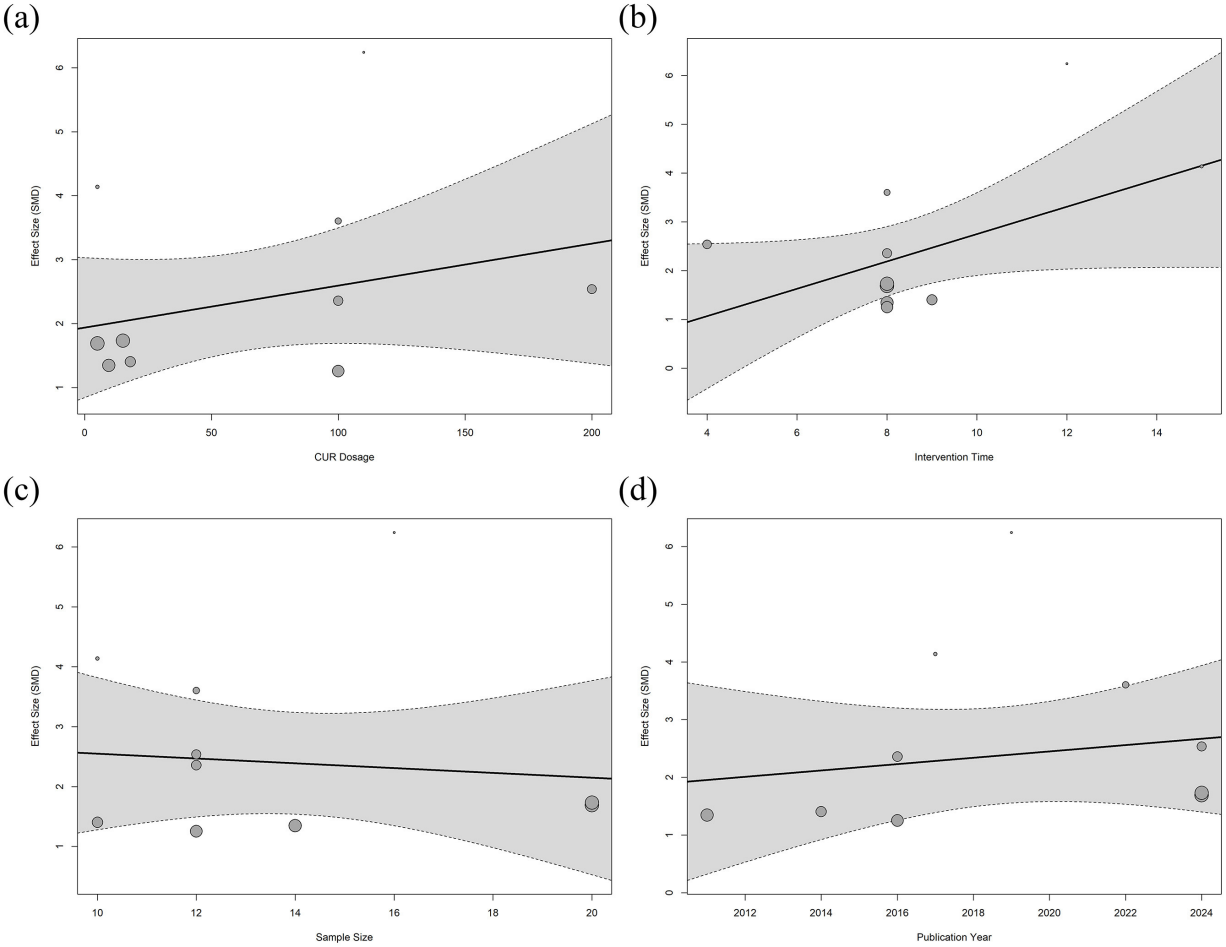
Meta-regression analysis of curcumin dose and treatment duration on bone mineral density. Meta-regression analysis of femoral BMD. **(a)** CUR intervention dose, **(b)** Intervention duration, **(c)** Sample size, and **(d)** Year of publication.

This study explored the effects of various potential moderating variables on the efficacy of BMD in animal models of osteoporosis through meta-regression analysis. Four variables were included in the regression analysis, including the intervention dose of CUR, intervention duration, sample size, and year of publication. The results of the regression model are shown in Figure 8. The meta-regression results showed that most of the moderating variables were not significantly correlated with the improvement of bone mineral density in osteoporosis (*P* > 0.05). Only the duration of the CUR intervention showed a marginal effect on the improvement of bone mineral density in osteoporosis (regression coefficient = 0.2801, 95% CI = −0.0165 to 0.5767, adjusted R^2^ = 26.47%), and the difference was not statistically significant (*P* = 0.0642). The regression model showed that the bone density of the osteoporosis animal model may improve slightly with the extension of the intervention time of the CUR (Figure 8b). However, the remaining adjusted variables were not statistically significant for improving osteoporosis bone density.

### 3.7 Sensitivity analysis

**Figure 9:**
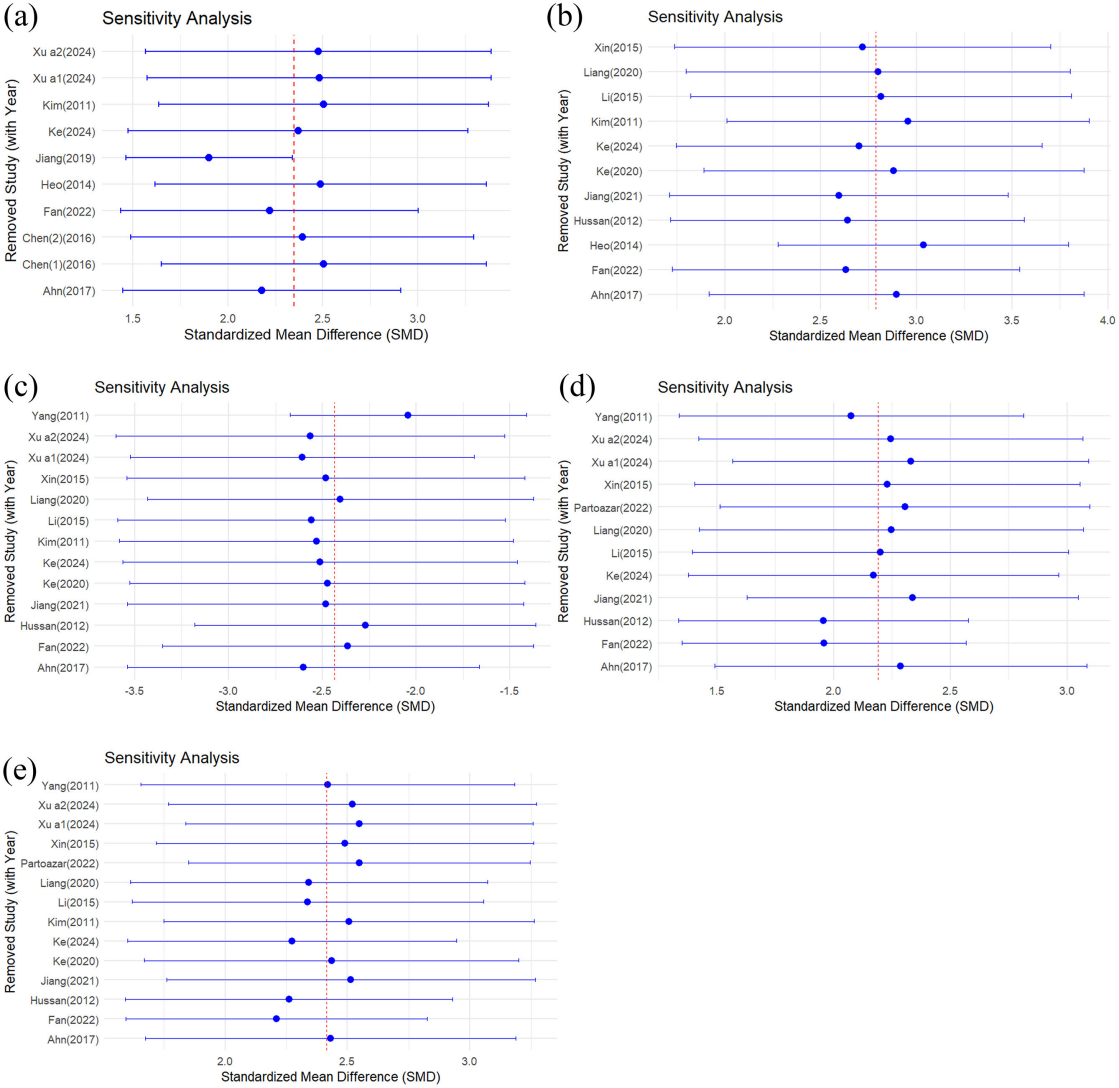
Sensitivity analysis of the effect of curcumin on bone mineral density and trabecular microstructure. **(a)** Femoral BMD, **(b)** BV/TV, **(c)** Tb.N, **(d)** Tb.Th, and **(e)** Tb.Sp.

A sensitivity analysis was conducted to evaluate the influence of individual studies on the combined results for femoral BMD and related bone trabeculae indicators (BV/TV, Tb.N, Tb.Th, and Tb.Sp). Each study was sequentially excluded using a stepwise method, and the combined effect values of the remaining studies were recalculated. The results showed that regardless of the exclusion of any individual study, the 95% confidence interval of the combined effect value did not change significantly (Figure 9), indicating that the analysis results were less dependent on a single study and the conclusions were highly robust. Specifically, the combined effect values for femoral BMD (Figure 9a), BV/TV (Figure 9b), Tb.N (Figure 9c), Tb.Th (Figure 9d), and Tb.Sp (Figure 9e) remained consistent after excluding any individual study, with no significant fluctuations. This result indicates that the improvement effect of CUR on bone density and trabecular structure in animal models of osteoporosis is highly stable and reliable.

### 3.8 Publication bias analysis

**Figure 10:**
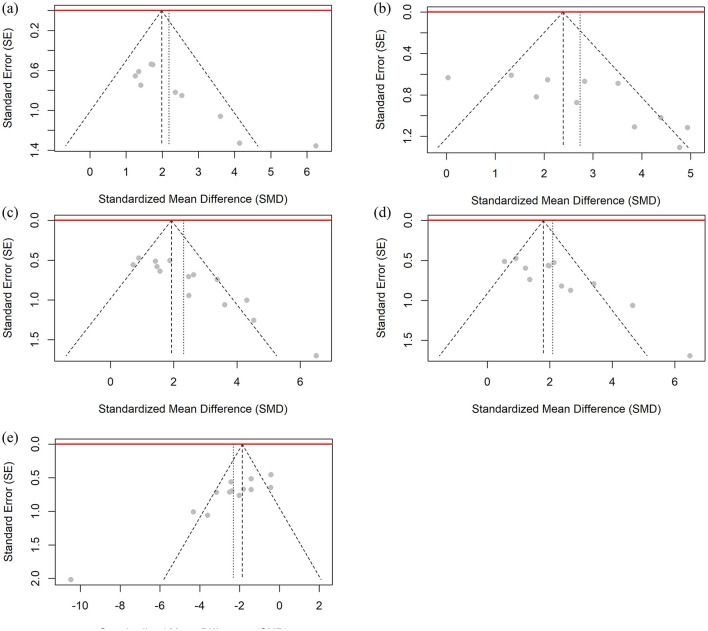
Funnel plot for publication bias assessment of BMD and bone microstructure data. **(a)** Femoral BMD, **(b)** BV/TV, **(c)** Tb.N, **(d)** Tb.Th, and **(e)** Tb.Sp.

This study used Egger regression tests to evaluate potential publication bias in the studies of femoral BMD, BV/TV, Tb.N, Tb.Th, and Tb.Sp. As shown in Figure 10, the Egger test results showed significant asymmetry in the funnel plots for the above outcome indicators, suggesting that there may be publication bias in the studies that included these outcome indicators.

To address potential publication bias in the outcome indicators, this study applied the Trim-and-Fill method for bias correction (46). The funnel plots for all outcome indicators can be found in the Supplementary material. This method identified and supplemented several potentially missing studies on the smaller side of the mean effect value. After correction, the number of included studies for each outcome increased, and the combined effect value decreased slightly from the value before correction. However, the corrected effect value remained statistically significant (e.g., femoral BMD corrected SMD = 1.7206, 95% CI: 0.7074–2.7338, *P* = 0.0009). This result indicates that the intervention effect of CUR on the osteoporosis animal model has high robustness, and that the significant effect is not entirely explained by publication bias, whether it is femoral BMD or other bone trabecular-related indicators (such as BV/TV, Tb.N, Tb.Th, and Tb.Sp).

### 3.9 GRADE evaluation

The quality of evidence for the outcome measures was assessed using the GRADE approach in this study. As shown in Table 4, the evidence quality was categorized as moderate (*n* = 2), low (*n* = 2), and very low (*n* = 7). Therefore, the overall quality of evidence for the outcome measures in this study is relatively low. The GRADE assessment indicates that the majority of the outcome measures are at high to very high risk of bias.

**Table 4:** GRADE evidence profile.

**Quality assessment**							**No of animals**		**Effect**		**Quality**	**Importance**
**No of studies**	**Design**	**Risk of bias**	**Inconsistency**	**Indirectness**	**Imprecision**	**Other considerations**	**Curcumin**	**Control**	**Relative (95% CI)**	**Absolute**		
**F-BMD (Better indicated by lower values)**												
10	Experimental studies	Serious^a^	Serious^b^	No serious indirectness	No serious imprecision	Reporting bias^f^	69	69	-	SMD 2.21 higher (1.52 to 2.9 higher)	⊕○○○ Very low	Critical
**T-BMD (Better indicated by lower values)**												
2	Experimental studies	Serious^a^	No serious inconsistency	No serious indirectness	Serious^d^	None	15	15	-	SMD 1.09 higher (0.3 to 1.87 higher)	⊕⊕○○ Low	Critical
**BV/TV (Better indicated by lower values)**												
11	Experimental studies	Serious^a^	Serious^b^	No serious indirectness	No serious imprecision	Reporting bias^f^	81	81	-	SMD 2.73 higher (1.84 to 3.63 higher)	⊕○○○ Very low	Important
**Tb.N (Better indicated by lower values)**												
14	Experimental studies	Serious^a^	Serious^b^	No serious indirectness	No serious imprecision	Reporting bias^f^	112	112	-	SMD 2.28 higher (1.65 to 2.91 higher)	⊕○○○ Very low	Important
**Tb.Th (Better indicated by lower values)**												
12	Experimental studies	Serious^a^	Serious^b^	No serious indirectness	No serious imprecision	Reporting bias^f^	97	97	-	SMD 2.08 higher (1.43 to 2.73 higher)	⊕○○○ Very low	Important
**Tb.Sp (Better indicated by lower values)**												
12	Experimental studies	Serious^a^	Serious^b^	No serious indirectness	No serious imprecision	Reporting bias^f^	96	96	-	SMD 2 lower (2.64 to 1.37 lower)	⊕○○○ Very low	Important
**Ca (Better indicated by lower values)**												
4	Experimental studies	Serious^a^	Serious^b^	No serious indirectness	Very serious^e^	None	28	28	-	SMD 0.47 higher (0.35 lower to 1.29 higher)	⊕○○○ Very low	Important
**P (Better indicated by lower values)**												
4	Experimental studies	Serious^a^	No serious inconsistency	No serious indirectness	Serious^d^	None	28	28	-	SMD 0.66 higher (0.11 to 1.21 higher)	⊕⊕○○ Low	Important
**CTX-1 (Better indicated by lower values)**												
4	Experimental studies	Serious^a^	No serious inconsistency	No serious indirectness	No serious imprecision	None	31	31	-	SMD 1.85 lower (2.49 to 1.22 lower)	⊕⊕⊕○ Moderate	Important
**OCN (Better indicated by lower values)**												
5	Experimental studies	Serious^a^	Very serious^c^	No serious indirectness	Serious^d^	None	36	36	-	SMD 1.6 higher (0.37 to 2.82 higher)	⊕○○○ Very low	Important
**TRAP-5b (Better indicated by lower values)**												
3	Experimental studies	Serious^a^	No serious inconsistency	No serious indirectness	No serious imprecision	None	21	21	-	SMD 2.16 lower (2.98 to 1.34 lower)	⊕⊕⊕○ Moderate	Important

GRADE Working Group grades of evidence: High quality: Further research is very unlikely to change our confidence in the estimate of effect. Moderate quality: Further research is likely to have an important impact on our confidence in the estimate of effect and may change the estimate. Low quality: Further research is very likely to have an important impact on our confidence in the estimate of effect and is likely to change the estimate. Very low quality: We are very uncertain about the estimate. ^a^Substantial risk of bias due to deficiencies in random sequence generation, allocation concealment, and blinding across the included studies. ^b^Moderate heterogeneity detected (*I*^2^ > 50 %, *p* < 0.05). ^c^High heterogeneity detected (*I*^2^ > 75 %, *p* < 0.05). ^d^Confidence interval is very wide, spanning at least two categories of effect size. ^e^Effect estimate extremely imprecise: the CI is very wide, crosses zero and covers multiple effect-size categories. ^f^Marked funnel-plot asymmetry; Egger's test *P* < 0.05, indicating possible publication bias.

## 4 Discussion

### 4.1 Potential application prospects of CUR in osteoporosis treatment

It is well known that OP is often referred to as the “silent killer,” and its main complication, osteoporotic fractures, causes significant morbidity and mortality worldwide. The prevention and treatment of OP remains a key issue that requires urgent attention in public health. Although traditional drug treatments have shown some efficacy, their side effects and long-term safety issues highlight the need to develop alternative therapies. Natural compounds derived from traditional plants (e.g., CUR) have attracted increasing attention due to their multi-target mechanism of action and relatively high safety profile. In recent years, the potential efficacy of CUR in treating OP has received widespread attention. However, the majority of studies on its anti-OP efficacy or mechanism of action have been confined to animal or cell experiments, which has somewhat hindered the clinical advancement of CUR. This study systematically evaluated the therapeutic potential of CUR in animal models of OP, providing key preclinical evidence to support the conduct of clinical trials.

In this meta-analysis, this study included 17 high-quality studies, including a total of 151 animal models of OP. The results showed that CUR significantly improved BMD (femoral BMD and tibial BMD), BV/TV, Tb.N, and Tb.Th, while significantly reducing Tb.Sp in animal models of OP. No significant changes in serum calcium concentrations were observed after CUR intervention. In addition, CUR significantly reduced serum CTX-1 and TRAP-5b concentrations while showing increased serum OCN and ALP levels. These results further support the potential role of CUR in improving OP pathology by regulating bone metabolic indicators. It is worth noting that the heterogeneity analysis in the meta-analysis showed that the results of BMD, BV/TV, Tb.N, Tb.Th, and Tb.Sp showed high heterogeneity. For this reason, subgroup analysis and meta-regression were performed in this study to explore the possible sources of heterogeneity. The results showed that factors such as treatment duration, year of publication, sample size, the dose of CUR and the species of animal models did not have a significant impact on the heterogeneity of the overall results. This suggests that the ameliorative effect of CUR on the OP model is relatively stable under different experimental conditions. This study provides preliminary evidence that CUR has a significant osteoprotective effect on the OP animal model through multiple mechanisms, which is manifested as promoting bone formation and inhibiting bone resorption. The results of this study provide important preclinical support for the clinical application of CUR as a potential anti-OP drug.

### 4.2 Strengths of the study

This is the first systematic review and meta-analysis to explore the therapeutic effects and potential mechanisms of CUR in an animal model of OP. This study has several notable advantages. First, this study adhered to the PRISMA guidelines for performing systematic reviews and meta-analysis, and two independent researchers completed all steps to reduce subjective bias in the research process and significantly improve the rigor of the study and the credibility of the results. Second, the literature included in this study is all high-quality animal experiments, and the research focuses on the single component of CUR, eliminating the interference of mixed derivatives, thereby providing a more accurate assessment of efficacy and an important theoretical basis for the future clinical development of CUR. Third, this study comprehensively evaluated multiple osteoporosis-related indicators (such as BMD, BV/TV, Tb.N, Tb.Th, and Tb.Sp), and combined the analysis of serum metabolic markers (such as CTX-1, TRAP-5b, and OCN), systematically revealing the comprehensive effects of CUR in promoting bone formation and inhibiting bone resorption. In addition, clearly defined research questions and strict inclusion criteria reduced bias in the selection of animal experiments, thereby improving the consistency and reliability of the research results. Finally, subgroup analysis and meta-regression were used to explore sources of heterogeneity. It was found that the duration of treatment, sample size, and type of animal model were not the main sources of heterogeneity, further supporting the stability of the efficacy of CUR. At the same time, the sensitivity analysis results showed that this study's conclusions are highly robust. In summary, this study clarifies the multi-level therapeutic effects of CUR on OP and provides a systematic theoretical basis and experimental evidence for future clinical studies on CUR.

### 4.3 Limitations and challenges

Although this study systematically evaluated the therapeutic effects of CUR on OP animal models and their potential mechanisms, some limitations should be noted. First, there were some differences in the methodological quality of the included studies. As can be seen from the results of the risk of bias assessment (Figure 3), although most studies showed low bias in terms of random sequence generation, baseline characteristic balance, and randomization grouping, there were significant deficiencies in the implementation of blinding, outcome assessment, and handling of incomplete data. For example, only two studies clearly used the random number table method (38, 45). Eight studies did not indicate whether the animals were randomly assigned (29, 30, 35, 39, 40, 42–44). And another seven studies mentioned randomization but did not describe the specific grouping method (31–34, 36, 37, 41), resulting in unclear risk of selection bias. In terms of the handling of incomplete data, seven studies were assessed as having a low risk (32, 34–36, 38, 41, 44), six studies as having an unclear risk (30, 31, 40, 42, 43, 45), and four studies as having a high risk (29, 33, 37, 39), suggesting that the lack of appropriate handling of missing data may have led to selective reporting bias. These issues affected the reliability and robustness of the study results. Second, this study had high heterogeneity for some key indicators (such as BMD, BV/TV, Tb.N, Tb.Th, and Tb.Sp). Although potential sources of heterogeneity were explored through subgroup analysis and meta-regression analysis, treatment duration, CUR dose, and animal model type did not fully explain the heterogeneity. This may be related to the diversity of experimental designs (e.g., experimental conditions, feeding environment, and baseline differences) and the incompleteness of the research reports, suggesting that other potential influencing factors that were not considered may have had a particular impact on the research results. In addition, the sample size calculation was not clearly reported in some studies (30, 31, 40, 42, 43, 45), which may lead to insufficient statistical power. Third, the choice of animal models may not fully simulate the complex pathological process of human OP. In particular, there may be species differences between animal models and humans regarding pathological characteristics and drug metabolism, which challenges the clinical translatability of the results. Finally, the statistical precision of the effect estimates may be affected due to the small sample size of some studies (32, 35, 36, 42, 44), especially in the study of serum metabolic markers (such as CTX-1, TRAP-5b, etc.). Although sensitivity analysis shows that the results are highly robust, the potential impact of small sample size and publication bias should not be ignored. Additionally, we formally assessed the certainty of evidence using the GRADE approach adapted for animal studies. Owing to risk of bias, inconsistency, and suspected publication bias, nine of the eleven pre-specified outcomes were downgraded to low or very-low certainty, and only CTX-1 and TRAP-5b achieved moderate certainty. These ratings indicate that the current animal data are hypothesis-generating rather than practice-changing and should therefore be interpreted with caution until confirmed by rigorously designed clinical trials. In summary, this study provides important preclinical evidence for the clinical application of CUR as a potential anti-OP drug. Nevertheless, future high-quality, large-sample prospective animal studies are required to further validate our findings and enhance the reliability and stability of the results.

### 4.4 Future research directions and application prospects of CUR

This study further consolidated the therapeutic prospects of CUR as a potential anti-osteoporosis drug in OP animal models through systematic meta-analysis. However, extrapolation from experimental animal studies to treating human diseases should be treated with caution, and the direction and strategy of future research is crucial to promote the clinical translation of CUR. First, the significant efficacy of CUR in OP animal models provides theoretical support for follow-up studies. Compared with other natural compounds, CUR showed a more comprehensive improvement effect, significantly increasing key bone structure parameters such as BMD, BV/TV, and Tb.N, and effectively reducing Tb.Sp. These findings indicate that CUR might have an anti-osteoporosis effect by enhancing the trabecular microenvironment to promote bone formation and inhibit bone resorption. However, the existing studies have not fully revealed the mechanism of action of CUR, especially its specific role in regulating bone metabolic signaling pathways such as OPG/RANKL and Wnt/β-catenin. Therefore, future studies should further combine molecular biology experiments to reveal the multi-target mechanism of CUR action and lay the foundation for its clinical translation. Secondly, the low bioavailability of CUR limits its potential for clinical application (47, 48). Studies have shown that CUR has low absorption and metabolic efficiency *in vivo*, often leading to limited bioavailability (49). Although some studies have explored the combination of CUR with other drug carriers (such as liposomes or nanocarriers) to improve its bioavailability and bone targeting (43, 50), the application of these techniques in OP treatment is still limited. Therefore, future drug development should focus on enhancing the bone-targeting therapeutic effect of CUR and maximizing its anti-osteoporosis potential through a rational drug delivery system. In addition, the dose-dependent effect of CUR needs to be further clarified. Current studies have not fully explored the therapeutic effect and safety of CUR at different doses. For example, lower doses may not significantly improve osteoporosis (51), while higher doses may pose potential toxicity risks (52). Therefore, further dose-response and long-term intervention studies are needed in future research to clarify the dosage of CUR for the treatment of OP and to evaluate its safety and long-term efficacy. It is noteworthy that critical appraisal of the nine available clinical trials revealed substantial heterogeneity in both the curcumin formulations employed and the definitions of outcome measures; only one study used a single-ingredient curcumin preparation that met the PICOS criteria of the present review. Accordingly, a methodologically robust clinical meta-analysis is not currently feasible. This evidence gap highlights the need for future randomized controlled trials with adequate statistical power that employ a standardized, Good Manufacturing Practice (GMP)–compliant single-ingredient curcumin formulation and utilize dual-energy X-ray absorptiometry (DXA) endpoints recommended by the International Society for Clinical Densitometry (ISCD) (53), thereby providing a rigorous bridge between pre-clinical research and clinical application. Finally, combining CUR with other natural active substances is an area of interest. Previous studies have shown that the synergistic effect of CUR with other natural active substances, such as Ligustrum lucidum (FLL) (54) or other bone metabolism-related factors (such as some miRNAs) (33, 55), may enhance its anti-OP effect. However, this hypothesis needs to be verified experimentally. Future studies could explore the combined application strategies of CUR with various bone metabolism regulators to optimize its therapeutic effect further and explore its potential value in comprehensive treatment regimens. In summary, this study provides important preclinical support for using CUR in treating OP and clarifies the key directions for future research on CUR. By further exploring its mechanism of action, optimizing drug delivery technology, and verifying combination therapy strategies, CUR is expected to have more significant clinical potential in the treatment of osteoporosis and open up new avenues for using natural compounds in the treatment of degenerative bone diseases.

### 4.5 Potential molecular mechanisms of CUR against osteoporosis

This study summarizes CUR's therapeutic effects in animal osteoporosis models through a meta-analysis and further explores its potential molecular mechanisms. The included literature shows that CUR exerts its anti-osteoporosis effect through multiple signaling pathways.

#### 4.5.1 OPG/RANKL signaling pathway

CUR exerts its anti-osteoporosis effect by upregulating osteoprotegerin (OPG) expression and inhibiting the level of nuclear factor κB receptor activator ligand (RANKL), thereby increasing the OPG/RANKL ratio, reducing osteoclastogenesis and activity, and protecting the structural integrity of bone trabeculae (33).

#### 4.5.2 Wnt/β-catenin signaling pathway

CUR promotes osteoblast differentiation and mineralization by reducing the inhibitory effect of oxidative stress on the Wnt/β-catenin signal, maintaining the dynamic balance of bone formation (31). In addition, CUR can also enhance the proliferation and differentiation of osteoblasts by downregulating the expression of Enhancer of Zeste Homolog 2 (EZH2) and relieving its inhibitory effect on the Wnt/β-catenin pathway (41). This mechanism provides a new molecular target for CUR's anti-osteoporosis effect.

#### 4.5.3 miR-365/MMP-9 regulatory axis

Studies have found that CUR can regulate specific microRNAs by upregulating miR-365 and inhibiting the expression of matrix metalloproteinase-9 (MMP-9), thereby reducing osteoclast activity and bone matrix degradation (33).

#### 4.5.4 NF-κB signaling pathway

CUR is a natural inhibitor of the Nuclear Factor kappa-light-chain-enhancer of activated B cells (NF-κB) signaling pathway (56). It may decrease the expression levels of crucial molecules in the NF-κB signaling pathway, including IκB-α degradation and p65 phosphorylation, thereby inhibiting osteoclast differentiation and activity and alleviating the adverse effects of inflammation and oxidative stress on bone formation (42, 45).

#### 4.5.5 MAPK signaling pathway

CUR plays an important role in the treatment of osteoporosis by regulating Extracellular Signal-Regulated Kinase (ERK), c-Jun N-terminal Kinase (JNK), and p38 in the Mitogen-Activated Protein Kinase (MAPK) signaling pathway. CUR inhibits the activation of RANKL signals, reduces the phosphorylation levels of ERK, JNK, and p38, and thus inhibits the differentiation and activity of osteoclasts (29). In addition, it has been found that CUR activates the ERK pathway by upregulating the expression of p-ERK1/2, a process that is associated with apoptosis. In particular, CUR significantly inhibited Dex-induced osteoblast apoptosis in a glucocorticoid (Dex)-induced osteoporosis model (35).

#### 4.5.6 TGF-β/Smad2/3 signaling pathway

CUR promotes osteoblast differentiation and matrix mineralization by enhancing the activity of the Transforming Growth Factor Beta (TGF-β)/Smad Family Member 2/3 (Smad2/3) signaling pathway, thereby improving the disordered trabecular bone structure caused by osteoporosis (40).

#### 4.5.7 Autophagy and JNK-BCL2-Beclin1 signaling pathway

CUR can regulate autophagy-related signaling pathways, including the JNK-BCL2-Beclin1 axis, promote intracellular waste removal, and protect osteoblasts from oxidative stress and inflammatory damage (44).

**Figure 11:**
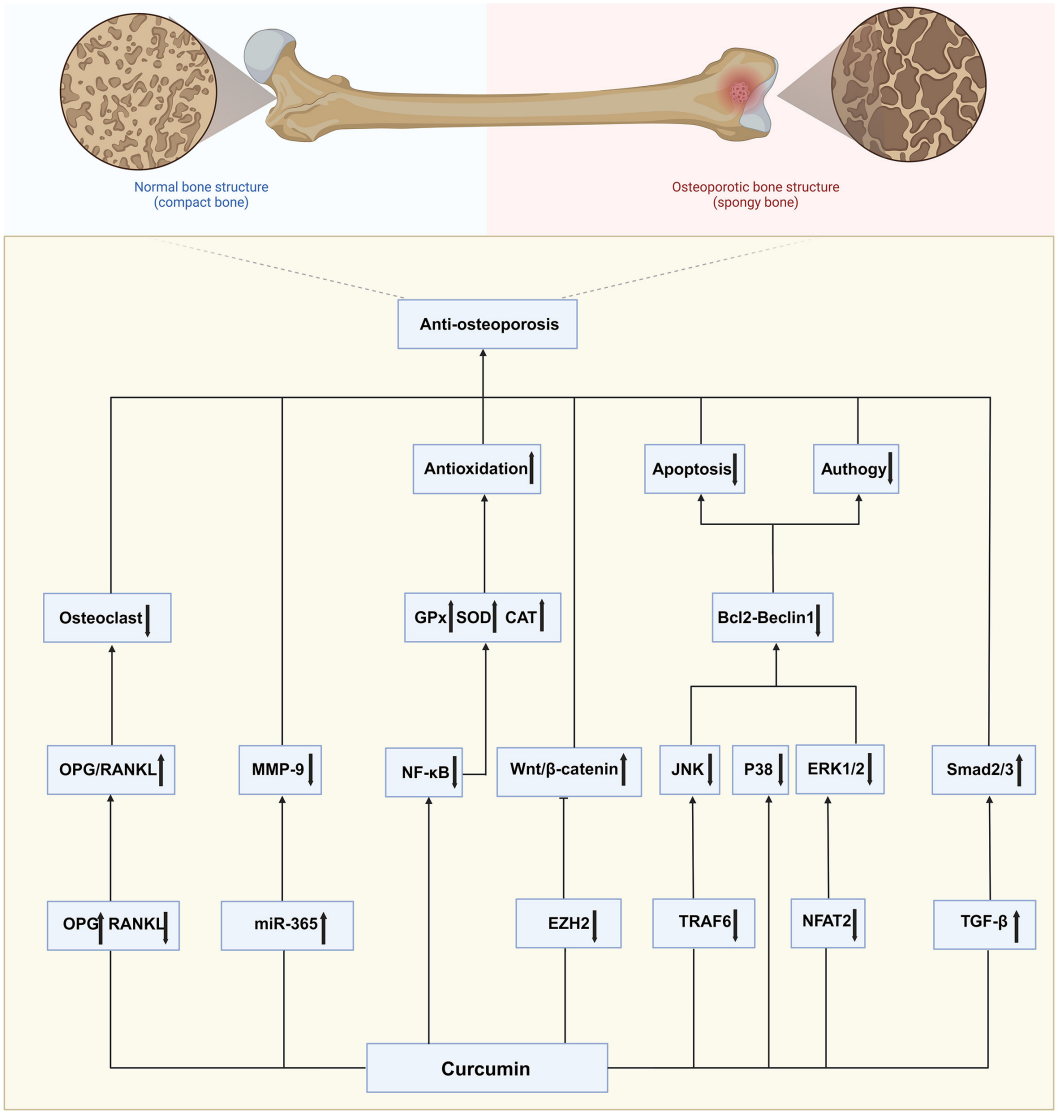
Curcumin's anti-osteoporotic effect: mechanisms and pathways.

The interaction of these signaling pathways suggests that CUR may exert a comprehensive protective effect on osteoporosis by regulating multiple molecular mechanisms. The results of this study not only reveal CUR's multi-target mechanism but also provide a theoretical basis and experimental evidence for its future clinical translation in the treatment of osteoporosis (Figure 11).

## 5 Conclusions

This study systematically reviewed and meta-analyzed the efficacy and potential mechanism of CUR in animal models of OP. The results showed that CUR could significantly improve BMD, bone trabecular microstructure (e.g., BV/TV, Tb.N, and Tb.Th), and bone metabolism indicators (e.g., ALP and CTX-1) while reducing Tb.Sp and osteoclast-related metabolic parameters (e.g., CTX-1 and TARP-5b) in animal models of OP. Mechanistic studies further revealed that CUR regulates the dynamic balance of bone remodeling by modulating multiple key signaling pathways, including the OPG/RANKL, Wnt/β-catenin, NF-κB, MAPK, and TGF-β/Smad2/3 pathways, to promote osteogenesis and inhibit osteoclast activity. In summary, CUR shows good prospects in the treatment of osteoporosis. Its multi-target mechanism and multi-level efficacy provide a strong theoretical basis and experimental support for its clinical development as a natural anti-osteoporosis drug. However, considering the high heterogeneity, risk of bias, and other limitations of the included studies, more high-quality, large-sample animal experiments and clinical trials are needed in the future to further deepen the understanding of the benefits of CUR in the treatment of OP and verify its safety and efficacy.
