# Supplementary material for: Comprehensive systematic review and meta-analysis on the therapeutic efficacy of curcumin in osteoporosis: unveiling mechanisms and preclinical evidence
Source: Front Nutr. 2025 May 21;12:1590256. doi: 10.3389/fnut.2025.1590256 (PMC12133540; doi:10.3389/fnut.2025.1590256)
Supplement: Supplementary file 1 [file Image_1.pdf]

frontiers

Supplementary Material

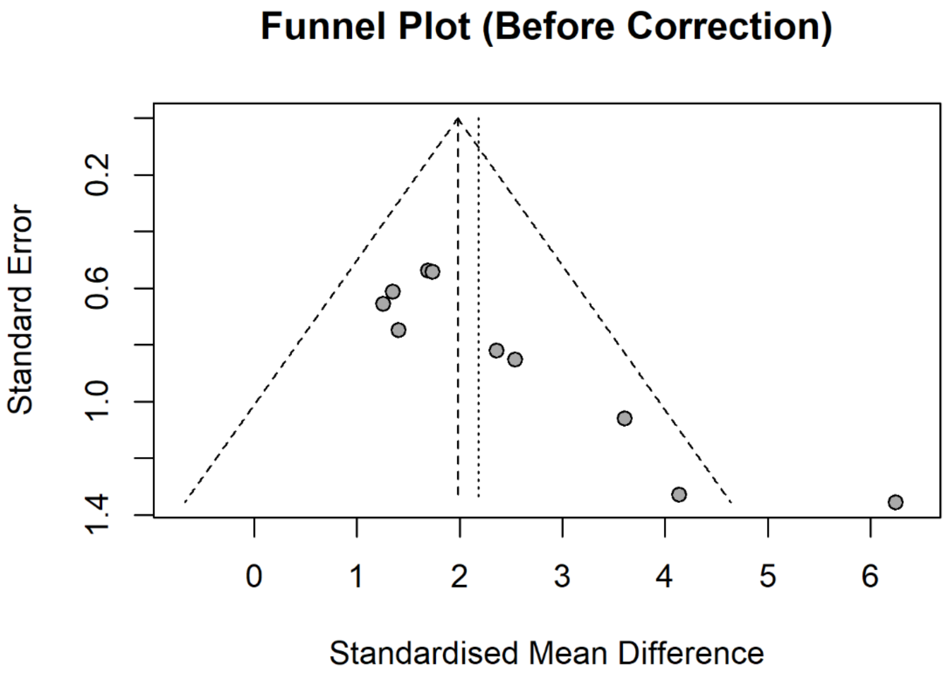

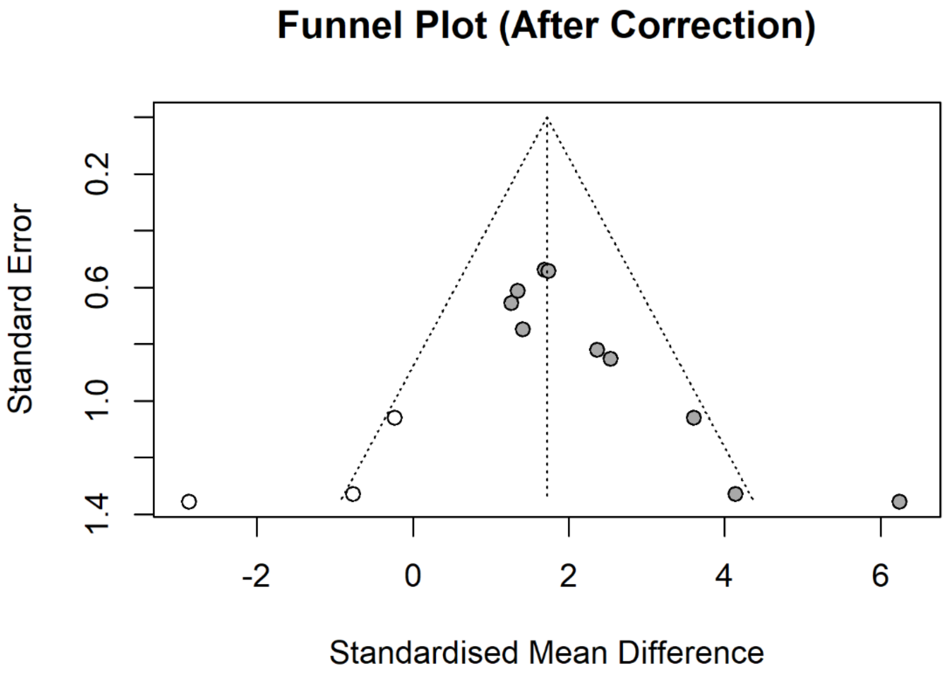

Supplementary Figure 1. Funnel Plot for Publication Bias Assessment of BMD Indicator. This figure shows the funnel plot assessing publication bias for the bone mineral density (BMD) indicator, before and after correction. The upper panel represents the funnel plot before correction, while the

lower panel shows the corrected version after applying the Trim-and-Fill method to adjust for potential publication bias.

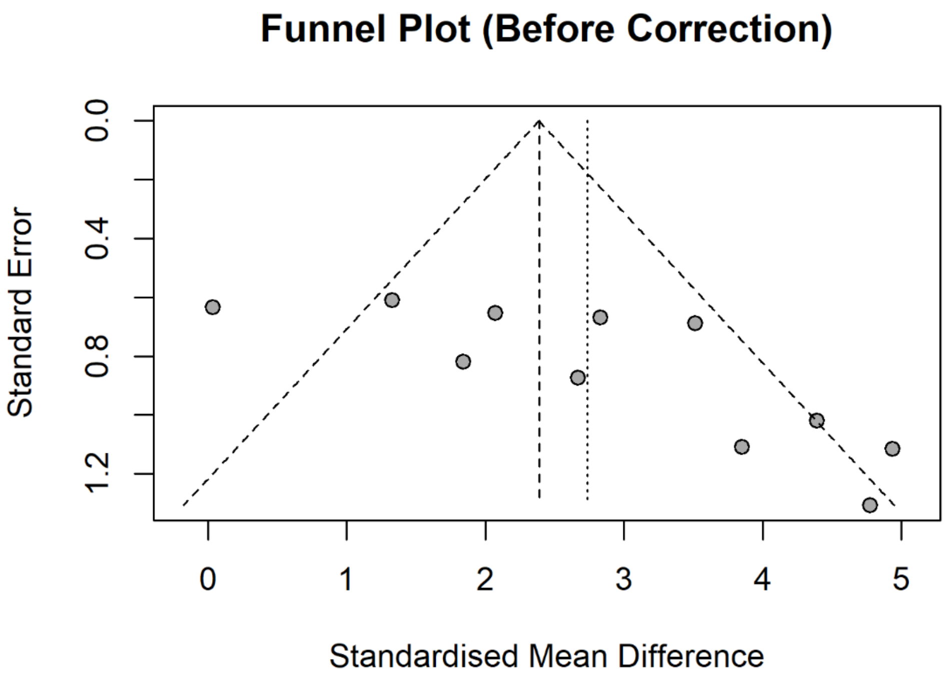

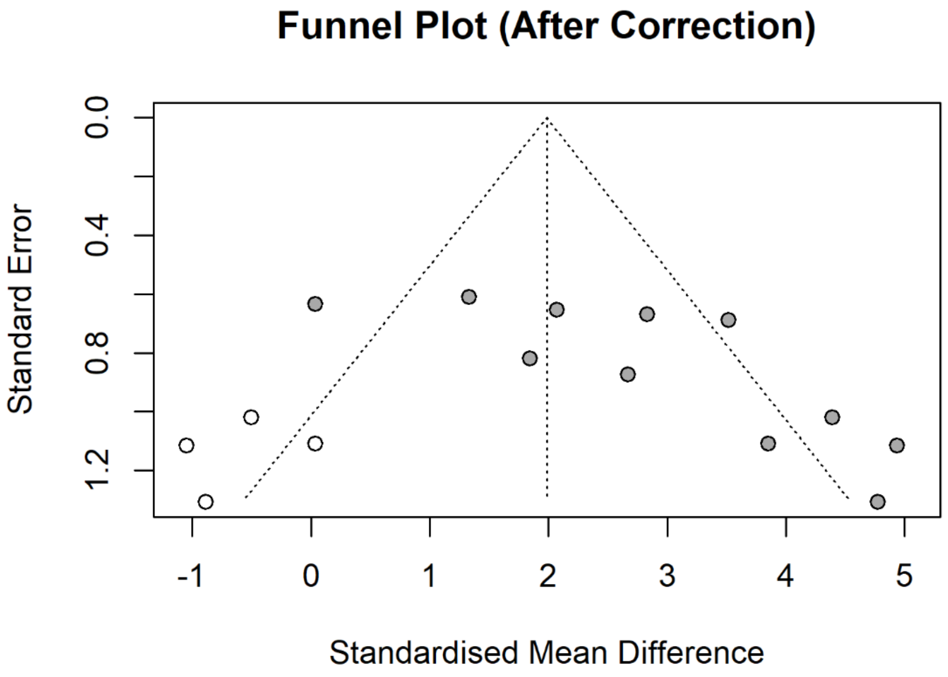

Supplementary Figure 2. Funnel Plot for Publication Bias Assessment of BV/TV Indicator. This figure shows the funnel plot assessing publication bias for the bone volume/total volume ratio

(BV/TV) indicator, before and after correction. The upper panel represents the funnel plot before correction, while the lower panel shows the corrected version after applying the Trim-and-Fill method to adjust for potential publication bias.

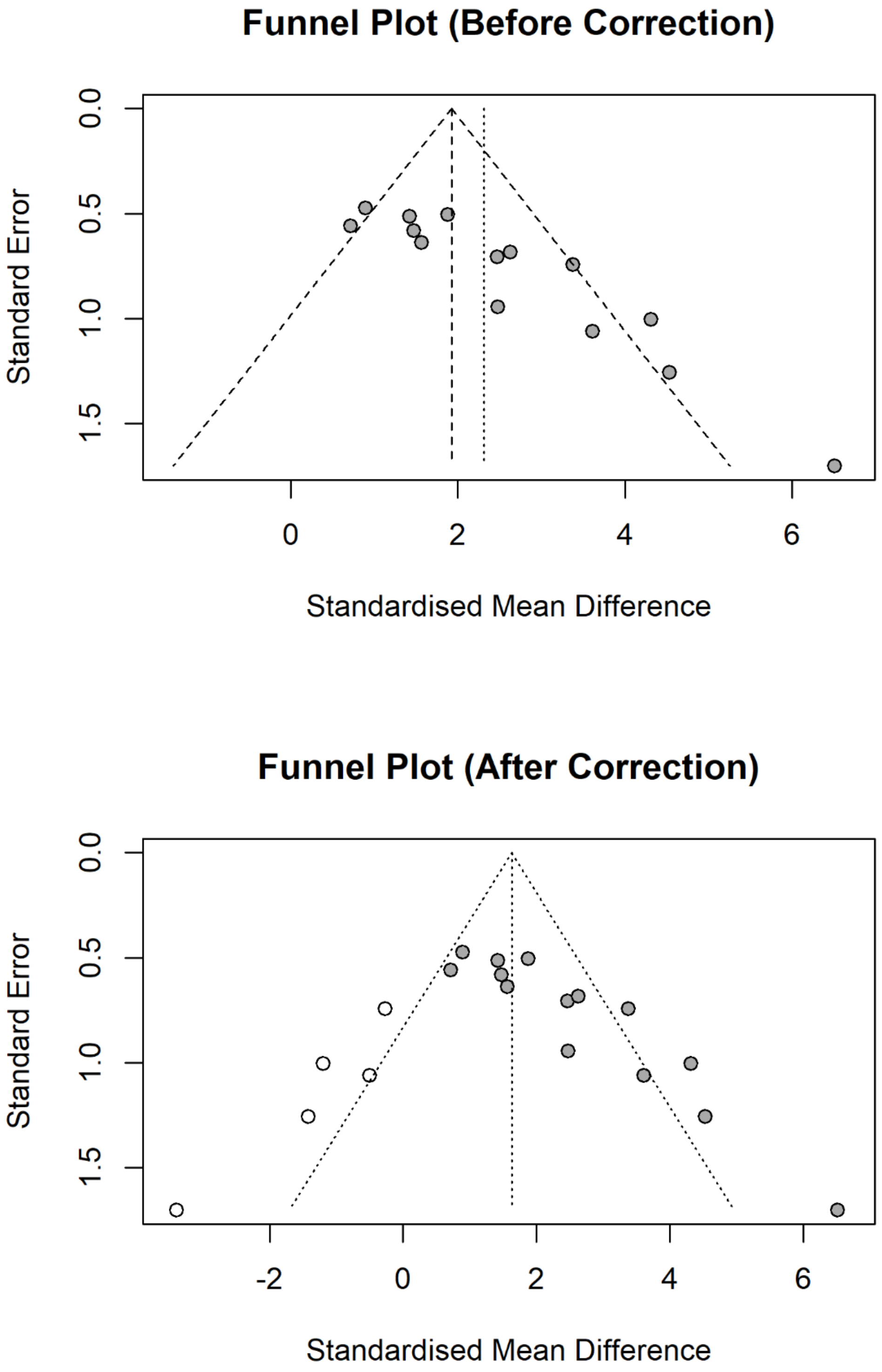

Supplementary Figure 3. Funnel Plot for Publication Bias Assessment of Tb.N Indicator. This figure shows the funnel plot assessing publication bias for the trabecular number (Tb.N) indicator, before and after correction. The upper panel represents the funnel plot before correction, while the lower panel shows the corrected version after applying the Trim-and-Fill method to adjust for potential publication bias.

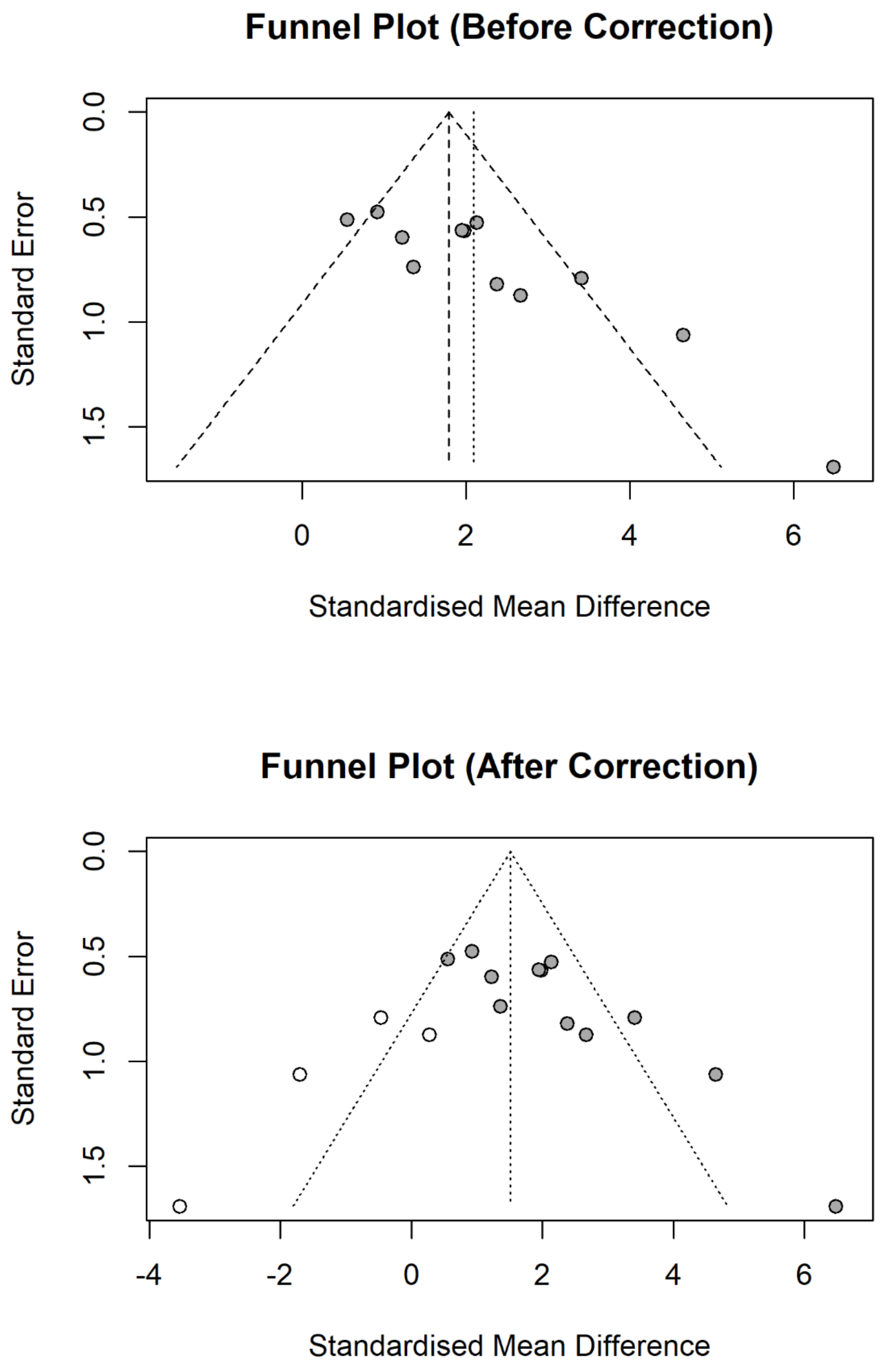

Supplementary Figure 4. Funnel Plot for Publication Bias Assessment of Tb.Th Indicator. This figure shows the funnel plot assessing publication bias for the trabecular thickness (Tb.Th) indicator, before and after correction. The upper panel represents the funnel plot before correction, while the

lower panel shows the corrected version after applying the Trim-and-Fill method to adjust for potential publication bias.

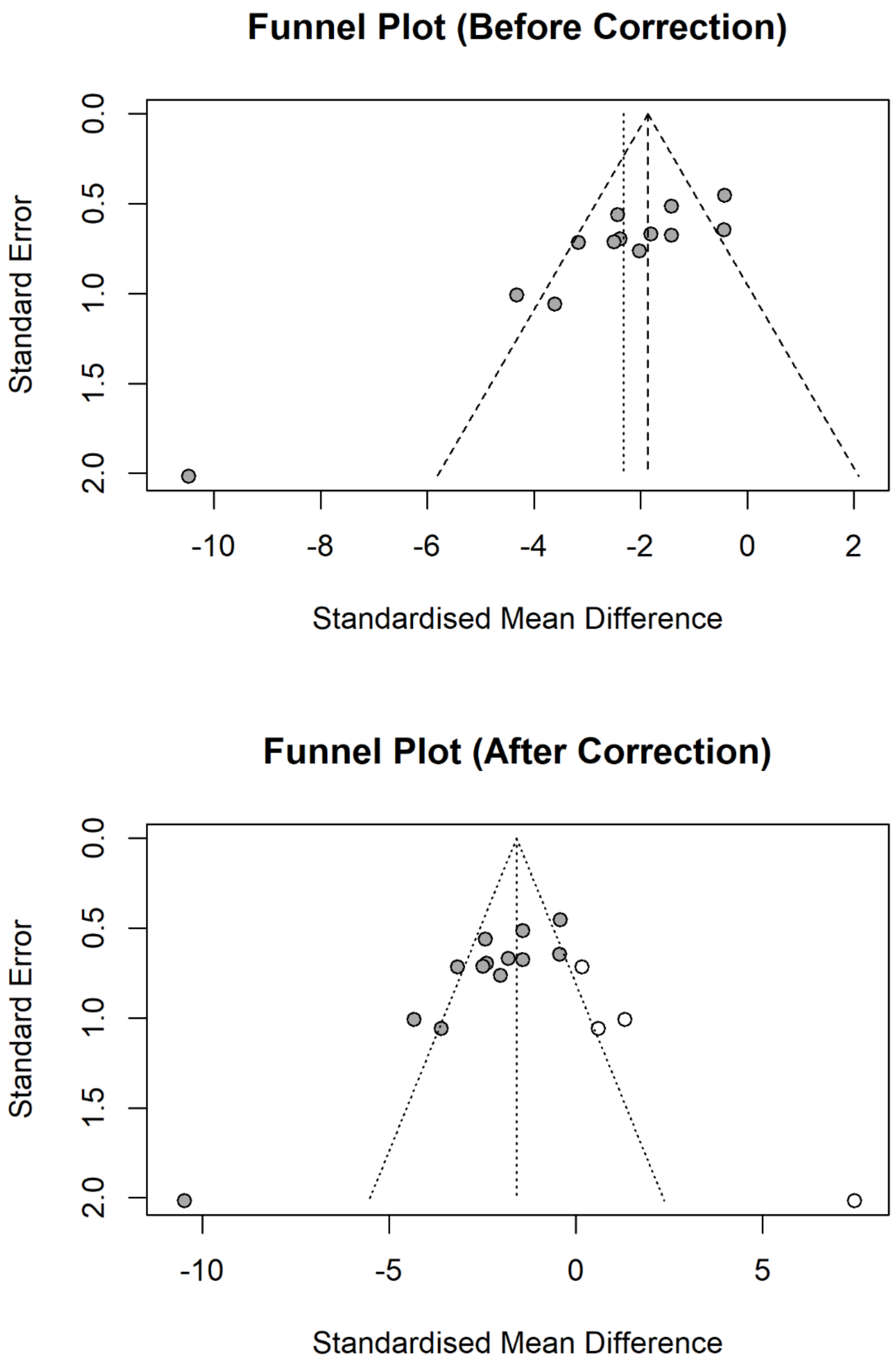

Supplementary Figure 5. Funnel Plot for Publication Bias Assessment of Tb.Sp Indicator. This figure shows the funnel plot assessing publication bias for the trabecular separation (Tb.Sp) indicator, before and after correction. The upper panel represents the funnel plot before correction, while the

lower panel shows the corrected version after applying the Trim-and-Fill method to adjust for potential publication bias.
